# The potential of using circulating tumour cells and their gene expression to predict docetaxel response in metastatic prostate cancer

**DOI:** 10.3389/fonc.2022.1060864

**Published:** 2023-01-16

**Authors:** Caitlin R. Davies, Tianyu Guo, Edwina Burke, Elzbieta Stankiewicz, Lei Xu, Xueying Mao, Glenda Scandura, Prabhakar Rajan, Karen Tipples, Constantine Alifrangis, Akhila Ganeshi Wimalasingham, Myria Galazi, Shanthini Crusz, Thomas Powles, Alistair Grey, Tim Oliver, Sakunthala Kudahetti, Greg Shaw, Daniel Berney, Jonathan Shamash, Yong-Jie Lu

**Affiliations:** ^1^ Centre for Cancer Biomarkers and Biotherapeutics, Barts Cancer Institute, Queen Mary University of London, London, United Kingdom; ^2^ Department of Cell Biology and the Second Affiliated Hospital, Zhejiang University School of Medicine, Hangzhou, China; ^3^ Central Biobank, Medical University of Gdansk, Gdansk, Poland; ^4^ Department of Urology, Zhongshan Hospital, Fudan University, Shanghai, China; ^5^ Centre for Cancer Cell and Molecular Biology, Barts Cancer Institute, Queen Mary University of London, London, United Kingdom; ^6^ Department of Urology, Barts Health National Health Service Trust (NHS), London, United Kingdom; ^7^ Division of Surgery and Interventional Sciences, University College London, London, United Kingdom; ^8^ University College London Hospitals, National Health Service (NHS) Foundation Trust, London, United Kingdom; ^9^ Department of Medical Oncology, Barts Health National Health Service (NHS) Trust, London, United Kingdom; ^10^ Centre for Experimental Cancer Medicine, Barts Cancer Institute, Queen Mary University of London, London, United Kingdom

**Keywords:** prostate cancer, circulating tumour cells, docetaxel, response prediction, biomarker, liquid biopsy, prognosis

## Abstract

**Background:**

Docetaxel improves overall survival (OS) in castration-resistant prostate cancer (PCa) (CRPC) and metastatic hormone-sensitive PCa (mHSPC). However, not all patients respond due to inherent and/or acquired resistance. There remains an unmet clinical need for a robust predictive test to stratify patients for treatment. Liquid biopsy of circulating tumour cell (CTCs) is minimally invasive, can provide real-time information of the heterogeneous tumour and therefore may be a potentially ideal docetaxel response prediction biomarker.

**Objective:**

In this study we investigate the potential of using CTCs and their gene expression to predict post-docetaxel tumour response, OS and progression free survival (PFS).

**Methods:**

Peripheral blood was sampled from 18 mCRPC and 43 mHSPC patients, pre-docetaxel treatment, for CTC investigation. CTCs were isolated using the epitope independent Parsortix^®^ system and gene expression was determined by multiplex RT-qPCR. We evaluated CTC measurements for post-docetaxel outcome prediction using receiver operating characteristics and Kaplan Meier analysis.

**Results:**

Detection of CTCs pre-docetaxel was associated with poor patient outcome post-docetaxel treatment. Combining total-CTC number with PSA and ALP predicted lack of partial response (PR) with an AUC of 0.90, p= 0.037 in mCRPC. A significantly shorter median OS was seen in mCRPC patients with positive CTC-score (12.80 vs. 37.33 months, HR= 5.08, p= 0.0005), ≥3 total-CTCs/7.5mL (12.80 vs. 37.33 months, HR= 3.84, p= 0.0053), ≥1 epithelial-CTCs/7.5mL (14.30 vs. 37.33 months, HR= 3.89, p= 0.0041) or epithelial to mesenchymal transitioning (EMTing)-CTCs/7.5mL (11.32 vs. 32.37 months, HR= 6.73, p= 0.0001). Significantly shorter PFS was observed in patients with ≥2 epithelial-CTCs/7.5mL (7.52 vs. 18.83 months, HR= 3.93, p= 0.0058). mHSPC patients with ≥5 CTCs/7.5mL had significantly shorter median OS (24.57 vs undefined months, HR= 4.14, p= 0.0097). In mHSPC patients, expression of *KLK2*, *KLK4*, *ADAMTS1*, *ZEB1* and *SNAI1* was significantly associated with shorter OS and/or PFS. Importantly, combining CTC measurements with clinical biomarkers increased sensitivity and specificity for prediction of patient outcome.

**Conclusion:**

While it is clear that CTC numbers and gene expression were prognostic for PCa post-docetaxel treatment, and CTC subtype analysis may have additional value, their potential predictive value for docetaxel chemotherapy response needs to be further investigated in large patient cohorts.

## Introduction

Prostate cancer (PCa) is the most frequently diagnosed cancer in Western males, accounting for 24% of all new cancers in 2018 (1). The effective first-line treatment for metastatic disease is androgen deprivation therapy (ADT), although after an initial response, progression to castration-resistant PCa (CRPC) occurs within 1-3 years (2). Adding docetaxel to ADT improves overall survival (OS) in metastatic (m)CRPC (3) and since 2014 as a result of the CHAARTED (4) and STAMPEDE (5) phase III trials, docetaxel has been used in combination with ADT as a first-line treatment for metastatic hormone-sensitive PCa (mHSPC) (3, 6). However, response to docetaxel is not universal due to inherent and/or acquired resistance. While numerous studies have investigated the underlying mechanisms and pharmacogenomic biomarkers of docetaxel resistance (7–11), there remains an unmet clinical need for new surrogate markers and a robust predictive test to stratify patients for treatment and develop personalised therapeutic approaches (12).

Tissue biomarkers representing cancer characteristics may help predict treatment outcome, but serial biopsies add morbidity and delay, and sampling of bone metastases is practically difficult. Furthermore, tissue biopsy fails to represent the entire cancer population due to intra-tumoural heterogeneity. In the case of therapy response prediction, markers that were detectable within the initial tissue biopsy sample are unlikely to truly represent the patient’s disease due to continuous tumour evolution at the molecular level. This is particularly important when considering second line therapies and beyond. As an alternative to tissue biopsy, liquid biopsy refers to the analysis of tumour biomarkers such as circulating tumour cells (CTCs), circulating tumour DNA (ctDNA), microRNA (miRNA) and extracellular vesicles (EVs) in peripheral blood or other body fluids. Liquid biopsies are minimally invasive, easily repeatable and can provide real time information of the heterogeneous tumour, providing a promising tool to overcome the limitations posed by tissue biopsy. Prostate-specific antigen (PSA) remains the standard serum biomarker for PCa diagnosis and progression, however with limited ability to predict therapeutic response (13).

CTCs are malignant cells that have gained an invasive phenotype, allowing them to shed from the tumour mass into the circulation where they travel to distant sites and form metastases (14). CTCs are unique amongst cancer biomarkers, as they provide a source of live tumour cells that carry molecular and biological information that may represent overall tumour burden and phenotypic characteristics present in both primary and metastatic sites. In addition to CTC enumeration, molecular profiling of enriched CTC populations or single CTCs provides a plethora of potentially clinically valuable markers of the metastatic process, disease status and predictors of patient individualised therapeutic response (15, 16). Therefore, CTC analysis may be a potentially ideal docetaxel response prediction biomarker.

Numerous studies have investigated various clinical applications of CTC enumeration and characterisation in PCa (17–22). Baseline CTCs have been shown to predict poor OS in patients with mCRPC (19, 23), which led to the FDA approval of CellSearch^®^ detected CTCs for advanced PCa prognosis (24). The MAINSAIL phase III trial of mCRPC patients treated with docetaxel found a significant association between baseline ≥5 CTCs/7.5ml of peripheral blood and poor OS, but not PSA response or Response Evaluation Criteria in Solid Tumors (RECIST) (25–27). The recent PROPHECY prospective multicentre study in patients with mCRPC undergoing treatment with enzalutamide or abiraterone followed by taxane chemotherapy, focused on the detection of CellSearch^®^ isolated CTCs expressing the androgen receptor splice variant, AR-V7. The study demonstrated that pre-treatment CTC AR-V7 status was independently associated with shorter progression free survival (PFS) and OS with abiraterone or enzalutamide, however men with AR-V7-positive disease still experienced clinical benefit from taxane chemotherapy (28). CTCs have also been investigated as predictive and prognostic biomarkers of clinical outcome, including mCRPC onset, in patients with mHSPC (17, 29–31), however to date there is limited information regarding their clinical utility in predicting docetaxel response in this patient cohort. Furthermore, the majority of studies to date have used epithelial epitope dependent isolation, missing a potentially important subpopulation of CTCs with epithelial negative phenotypes following epithelial-mesenchymal transition (EMT) during cancer cell invasion and metastatic spread. Previous research from ourselves and others has demonstrated that CTCs that are undergoing EMT, or those that have a fully mesenchymal phenotype have significant value as biomarkers of increased metastatic tumour burden (32), and disease progression (33, 34). Moreover, EMT is increasingly recognised as an important mechanism that drives inherent and acquired resistance to chemotherapies (35), including docetaxel (36). As such, exclusion of CTCs with epithelial negative phenotypes limits the detection of genes which might be developed into novel predictive biomarkers of docetaxel response, which may facilitate patient personalised treatment stratification.

We previously used the cell size and deformability based Parsortix^®^ CTC isolation system, which was recently FDA approved, to detect CTCs with epithelial, mesenchymal and intermediate phenotypes, and demonstrated their biomarker potential in different clinical scenarios (32, 37, 38). In this study we used Parsortix^®^ to capture pre-docetaxel treatment CTCs, evaluating CTC subtypes and their gene expression as biomarkers of docetaxel response in order to identify CTC markers with clinical value for the management of advanced PCa patients. Our strategy combined epitope independent CTC isolation for enumeration and molecular characterisation using multiplex RT-qPCR for a targeted panel of genes. We demonstrate the potential of analysing multiple CTC subtypes and their gene expression as predictive and prognostic biomarkers in both mCRPC and mHSPC patients.

## Methods

### Patients

Between January 2015 and January 2020, 18 mCRPC and 43 mHSPC patients were recruited with informed consented at St Bartholomew’s Hospital, Barts Health NHS, London, UK. Clinical characteristics for individual patients are shown in **Supplementary Table 1**. Peripheral blood samples were collected into EDTA tubes ≤2 months before commencing 6 cycles of docetaxel. Patients with mHSPC had started initial hormone-therapy <3 months before blood collection. Patients received CT and bone scans before and after treatment. Serum PSA, ALP, and LDH were measured together with CTC sampling. Radiological response assessment was based on RECIST criteria (25): (1) complete response (CR): disappearance of all target lesions; (2) partial response (PR): at least 30% decrease in the sum of the longest diameter of target lesions, taking as reference the baseline since treatment started; (3) progressive disease (PD): at least 20% increase in the sum of the longest diameter of target lesions, taking as reference the baseline since treatment started; (4) stable disease (SD): neither sufficient shrinkage to qualify for PR nor sufficient increase to qualify for PD. Assessments of response by bone scan were classified as follows: (1) CR: disappearance of all bone metastasis; (2) PR: a decrease in number, extent or intensity of bone lesions was detected; (3) PD: appearance of new bone lesion(s) and/or apparent enlargement of the bone metastases; (4) SD: little or no change in the number, extent or intensity of bone metastases was observed. PSA progression was defined as two consecutive rises above PSA nadir at least two weeks apart. CTC measurements were also investigated for their ability to prognose OS and PFS outcomes.

### Cell lines

The docetaxel-resistant human PCa cell line PC3-D12 and the sensitive counterpart PC3-Ag, were gifted by A.J. O’Neill (10), University College Dublin. PC3-D12 cells were treated every 4 weeks with 12 nM docetaxel in order to maintain resistance. The cells were maintained in RPMI-1640 medium supplemented with 10% foetal bovine serum and 2 nM L-glutamine (Invitrogen, Waltham, Massachusetts, Unites States).

### CTC isolation, enumeration and characterisation

CTCs were isolated from 7.5 mL of whole blood using the Parsortix^®^ (Angle Plc, Guildford, UK) isolation system and identified for CTC enumeration using four-colour immunofluorescence as previously described (32, 37). Briefly, 7 mL of blood was transferred to 50 mL LeucoSep tubes (Greiner Bio-One, Frickenhausen, Germany) with 15.3 mL of Ficoll-Paque Plus (GE Healthcare, Chicago, Illinois, Unites States) and centrifuged at 1000 g for 15 min with the break off at room temperature to recover the peripheral blood mononuclear cell (PBMC) fraction. The PBMC fraction along with the plasma above the fit of the LeucoSep tube was removed into a new 50 mL falcon tube and pelleted at 200 g for 8 min at room temperature. The pellet was then re-suspended in 4.5 mL of isolation buffer (PBS containing 1% BSA and 2 nM EDTA) and added back to the remaining 0.5 mL of whole blood and loaded onto the Parsortix^®^ for CTC isolation. Once samples are loaded, cells are separated based on cell size and deformability according to a pre-set programme PX-S99F that uses 6.5 µm-gap cassette and 99 mbar pressure for isolation. Cells were then harvested using a pre-set programme and transferred onto glass slides for downstream analysis. All blood samples were processed within 4 hrs of collection. Slides were stained using mouse monoclonal PE-conjugated anti-CD45 (Miltenyi Biotec, Bergisch Gladbach, Germany), mouse monoclonal FITC-conjugated anti-Cytokeratin (Miltenyi Biotec, Bergisch Gladbach, Germany), Alexa Fluor 647-conjugated anti-Vimentin (Abcam, Cambridge, UK), and counterstained using SlowFade gold antifade mountant with DAPI (Life Technologies, Carlsbad, California, United States). CTCs were identified as Cytokeratin (CK)+/Vimentin (VIM)−/CD45- (epithelial-CTCs), CK+/VIM+/CD45- (EMTing-CTCs) and CK-/VIM+/CD45- (mesenchymal-CTCs). Patients with ≥1 epithelial-CTC and/or ≥1 EMTing-CTC and/or ≥4 mesenchymal-CTCs were defined as CTC-score ‘positive’ using our previously established definition based on the analysis of healthy control blood samples (32)

### CTC RNA extraction and gene expression analysis

CTCs were isolated from a separate 7.5 mL of whole blood using the Parsortix^®^ and collected into a 1.5 mL low-retention eppendorf. Total RNA was extracted using miRNeasy micro kit (Qiagen, Hilden, Germany) following manufacturer’s instructions but eluted with a final volume of 11.5 μL. The total 11.5 µL of RNA extracted from CTCs was mixed with 0.5 µL of random primers and denatured at 65 °C for 5 min. After incubation for 5 min on ice, 4 µL of first strand buffer, 2 µL of 0.1 M DTT, 1 µL of 10 mM dNTPs (Roche, Basel, Switzerland), 0.5 µL of water and 0.5 µL of Superscript II (Thermo Fisher Scientific, Waltham, Massachusetts, Unites States) were added and cDNA synthesis was performed at 42 °C for 2 hrs, followed by enzyme inactivation by heating at 70 °C for 15 mins. Multiplex RT-qPCR was performed by Barts and the London Genome Centre using BioMark HD system (Fluidigm Corporation, South San Francisco, California, United States). 96.96 Dynamic Array Integrated fluidic circuit (IFC) was used to test expression levels of 32 assays in triplicates within one reaction plate. A list of TaqMan probes (Applied Biosystems, Massachusetts, Unites States) used are shown in **Supplementary Table 2**. The brief workflows were as follows: (1) pooling the TaqMan assays. Combine equal volumes of each 20X TaqMan Gene Expression assays in a 0.5 mL microcentrifuge tube, up to 100 μL in total. Dilute the pooled assays using DNA Suspension Buffer (10mM Tris, pH 8.0, 0.1 mM EDTA) so that each assay is at a final concentration of 0.2X. (2) Combine 2.5 μL of TaqMan^®^ PreAmp MasterMix (Life Technologies, Carlsbad, California, Unites States), 1.25 μL of pooled assay mix and 1.25 μL of cDNA to make the final sample mixture in each aliquot. (3) Place reaction tubes in the thermal cycler and cycle as (95 °C for 10 minutes followed by 14 cycles of 95 °C for 15 secs and then 60 °C for 4 mins). Only the targets of interests are amplified and this results in small amount of cDNA being amplified equally without introducing bias. Following pre-amplification, the samples were diluted 1:5 (v/v) in DNA suspension buffer. Reactions were then assayed using Dynamic Arrays prepared as instructed by the manufacturer. PCR was performed with 40 cycles of reactions.

### Gene panel selection

Two microarray expression profile data sets (GSE36135 (39), GSE33455 (11)) were downloaded from the Gene Expression Omnibus (GEO) database (http://www.ncbi.nlm.nih.gov/geo), which are based on the GPL571 Affymetrix Human Genome U133A 2.0 Array [HG_U133A_2] and GPL570 Affymetrix Human Genome U133 Plus 2.0 Array [HG-U133_Plus_2], respectively. The original Series Matrix data files were analysed with GEO2R (using the GEOquery and limma R packages from Bioconductor (http://www.bioconductor.org/) to identify differentially expressed genes (DEGs) in each of the paired docetaxel-resistant and docetaxel-sensitive cell lines in the datasets. A DEG was considered to be significant according to the following criteria: Fold-change (FC) >2 and false discovery rate (FDR) <0.05. Genes that were not upregulated in ≥2 DOC resistant cell lines were excluded to control for random variance in gene expression. Additionally, we identified reported PCa-specific and/or docetaxel-resistance related genes by literature search, and the two gene lists were combined to form a test gene panel. To select for genes suitable for CTC analysis, the genes were searched in The Genotype-Tissue Expression Portal V7 database for their expression in prostate and whole blood. Genes were selected based on their relative high expression in the prostate and low/no expression in whole blood. Candidate gene expression was subsequently validated in a panel of PCa cell lines and PBMC samples from five PCa biopsy negative males.

### Statistical analysis

Mann-Whitney U test was applied to assess differences in clinical characteristics between patient groups. Data were shown as median (interquartile range [IQR]). Spearman’s rank correlation was used to assess associations of CTC counts and gene expression with concurrent PSA, ALP, LDH levels and OS/PFS. A combined risk score (CRS) was computed as the linear predictor of the fitted bivariate logistic model with PSA, ALP, CTC-score, total-CTC number and KLK2 count as only predictors (as CRS = a * Y + b * X…, where the values of ‘a’ and ‘b’ are the estimated log odds ratios). Survival curves were generated using the Kaplan-Meier method and compared using the logrank test. Bonferroni correction method was applied to adjust p values (p_adj_) for multiple testing. RT-qPCR 2^-ΔΔCt^ was used to compare mRNA expression levels in patient samples. Receiver operating characteristic (ROC) curve analysis was used to evaluate prediction values. Follow-up time started on the date of administration of the first docetaxel dose. Observations were censored on the date of last follow-up.

Statistical analyses were performed using GraphPad Prism 9. All tests were two-sided with p values of <0.05 considered statistically significant.

## Results

### Patient characteristics and association of pre-docetaxel CTC measurements with clinicopathological data

**Figure 1:**
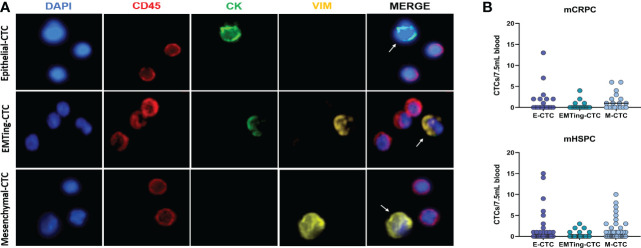
Detection of three subtypes of CTCs in PCa patient samples. **(A)** Three distinct CTC subtypes were identified by immunofluorescence in patient blood samples. Top: One CK+/VIM-/CD45- epithelial-CTC adjacent to two CD45+ leucocytes. Middle: One CK+/VIM+/CD45- EMTing-CTC adjacent to three CD45+ leucocytes. Bottom: One CK-/VIM+/CD45- mesenchymal-CTC adjacent to two CD45+ leucocytes. **(B)** Individual CTC numbers in each mCRPC and mHSPC patient sample, respectively. Median CTCs number per 7.5mL of blood is shown. Abbreviations: CK, Cytokeratin; VIM, Vimentin.

Clinical characteristics and CTC counts are summarised in **Table 1**. At least one CTC was detected in 12/18 (67%) of mCRPC patients, 50% of patients had a positive CTC-score (≥1 epithelial-CTC and/or ≥1 EMTing-CTC and/or ≥4 mesenchymal-CTCs) and the median total CTC count was 1.5 (interquartile range= 0-5.8). In mHSPC patients, ≥1 CTC was detected in 26/43 (60%), 51% of patients had a positive CTC-score and the median total CTC count was 1 (interquartile range = 0-4.3). **Figure 1A** shows an example of immunofluorescence staining for three CTC subtypes. **Figure 1B** shows individual CTC subtype counts in mHSPC and mCRPC patients. No significant differences were found between CTC subtype numbers.

**Table 1:** Summary of clinical characteristics and CTC enumeration for metastatic PCa patients.

	n	mCRPC	n	mHSPC
Age at pre-docetaxel, y				
Mean ± SD	18	73 (66.5-75.8)	43	68 (63-73)
PSA at diagnosis, ng/mL				
Median (IQR)	14	21 (14.5-61.5)	39	54 (18.5-344.6)
Biopsy GS, n (%)				
7		8 (44)		6 (14)
>7		9 (50)		28 (65)
unknown		1 (6)		9 (21)
Pre-docetaxel PSA, ng/mL				
Median (IQR**)**	18	54.1 (12.9-111.8)	42	15.6 (3.6-50.1)
Pre-docetaxel ALP, U/L				
Median (IQR)	16	88 (74.8-387.5)	36	104 (70-328)
Pre-docetaxel CTC-score, n (%)				
Positive		9 (50)		22 (51)
Negative		9 (50)		21 (49)
Pre-docetaxel total CTC, n				
Median (IQR)	18	1.5 (0-5.8)	43	1 (0-4.3)
Pre-docetaxel Epithelial-CTC, n				
Median (IQR**)**	18	0 (0-2)	43	0 (0-1)
Pre-docetaxel EMTing-CTC, n				
Median (IQR)	18	0 (0-0)	43	0 (0-0)
Pre-docetaxel Mesenchymal-CTC, n				
Median (IQR)	18	1 (0-2.25)	43	0 (0-2)

mCRPC, metastatic castration-resistant prostate cancer; mHSPC, metastatic hormone-sensitive prostate cancer; IQR, interquartile range; PSA, prostate specific antigen; GS, Gleason score; SD, standard deviation.

We subsequently investigated the relationship between CTCs and clinicopathological data. Spearman’s correlation was performed between CTCs and serum PSA and ALP, results are shown in **Table 2**. In mCRPC patients, serum PSA was significantly correlated with total-CTC (ρ= 0.51, p= 0.032), epithelial-CTC (ρ= 0.51, p= 0.030), EMTing-CTC numbers (ρ= 0.53, p= 0.024) and positive CTC-score (ρ= 0.68, p= 0.0021). Serum ALP was significantly correlated with total-CTC (ρ= 0.51, p= 0.046) and epithelial-CTC numbers (ρ= 0.62, p= 0.012). In mHSPC patients, serum ALP was significantly correlated with mesenchymal-CTC numbers (ρ= 0.34, p= 0.044), however no other significant correlations were observed.

**Table 2:** Spearman’s correlation between CTCs and serum PSA and ALP.

	PSA	ALP
	*Spearman’s ρ (p-value)*	
mCRPC patients		
Total-CTCs	**0.51 (0.032)**	**0.51 (0.046)**
Epithelial-CTCs	**0.51 (0.030)**	**0.62 (0.012)**
EMTing-CTCs	**0.53 (0.024)**	0.38 (0.15)
Mesenchymal-CTCs	0.22 (0.37)	0.27 (0.31)
CTC-score	**0.68 (0.0021)**	**0.45 (0.091)**
PSA	–	–
ALP	0.35 (0.19)	–
mHSPC patients		
Total-CTCs	-0.017 (0.92)	0.27 (0.11)
Epithelial-CTCs	0.14 (0.37)	0.080 (0.65)
EMTing-CTCs	0.066 (0.68)	-0.032 (0.86)
Mesenchymal-CTCs	-0.047 (0.77)	**0.34 (0.044)**
CTC-score	-0.006 (0.97)	0.21 (0.23)
PSA	–	–
ALP	**0.35 (0.044)**	–

bold black numbers, significant results

### Correlation of pre-docetaxel CTCs with RECIST response post docetaxel treatment

**Figure 2:**
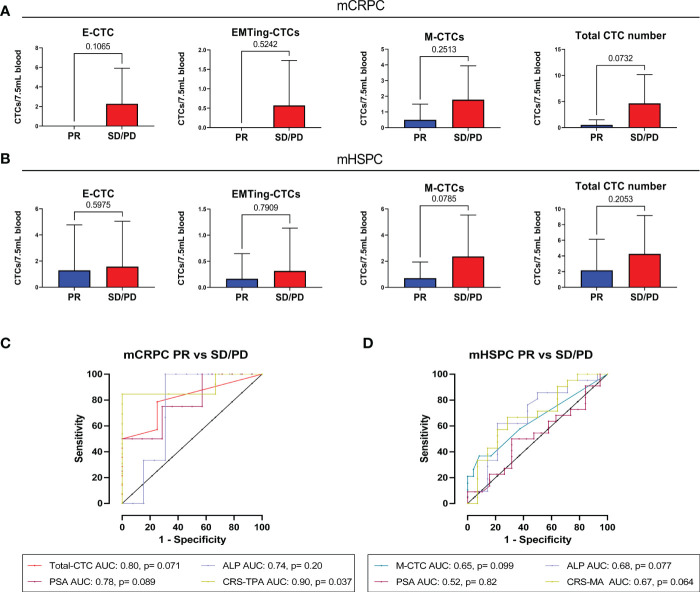
The relationship between pre-docetaxel CTCs and initial RECIST response. **(A)** Number of CTC subtypes and total-CTC numbers in mCRPC patients with PR or, SD and PD combined. Epithelial and EMTing-CTCs were not detected in patients with PR, although they did not significantly differentiate from patients with SD/PD, p=0.1065 and p=0.7907, respectively. Mesenchymal CTCs were detected in a small number of patients with PR but the majority were detected in patients with SD/PD (p=0.2513). Total CTC number trended towards a significant difference between patients with PR and SD/PD p=0.0732. **(B)** Number of CTC subtypes and total-CTC numbers in mHSPC patients with PR or, SD and PD combined. There was no significant difference in the numbers of epithelial-CTCs (p=0.5975), EMTing-CTCs (p=0.7909) and total-CTCs (p=0.2053) detected between patients with PR and SD/PD. However, mesenchymal-CTC numbers trended towards a significant difference between the patient outcome groups (p=0.0785). **(C)** For prediction of immediate tumour response in mCRPC patients, total-CTCs had an area under the curve (AUC) of 0.80, p= 0.071, PSA had an AUC of 0.78, p= 0.089 and ALP had an AUC of 0.74, p= 0.20. A combined risk score combining all three variables was calculated as CRS-TPA= 0.7414*Total-CTC number + 0.02909 *PSA + 0.01423*ALP, which increased the AUC to 0.90, p=0.037. **(D)** For prediction of immediate tumour response in mHSPC patients, mesenchymal-CTCs had that highest AUC of 0.65, p= 0.099, PSA had an AUC of 0.52, p= 0.82 and ALP an AUC of 0.68, p= 0.77. A combined risk score combining all mesenchymal CTC numbers and ALP was calculated as CRS-MA= 0.3599 * mesenchymal-CTC number + 0.002037 * ALP, resulted in an AUC of 0.69, p= 0.064.

To assess if CTCs could predict radiological response to docetaxel following treatment cycles, we compared CTC numbers between partial response (PR), stable disease (SD) or progressive disease (PD) groups (**Supplementary Table 1**), and performed ROC analysis. Due to the limited sample size, we combined patients who had SD or PD at the end of docetaxel treatment into one group. While there were no significant differences in CTC numbers between patients with PR and SD/PD in this small cohort, trends were observed. In mCRPC patients with PR, limited mesenchymal-CTCs and no epithelial- and EMTing-CTCs were detected. Total-CTCs trended towards a significantly lower number in patients with a PR (p= 0.073) compared to those with SD/PD (**Figure 2A**) with an AUC of 0.80, p= 0.071 in predicting SD/PD (**Figure 2C**). In comparison, serum PSA had an AUC of 0.78, p= 0.089, and ALP had an AUC of 0.74, p= 0.20 (**Figure 2C**). In order to improve our ability to predict radiological response to docetaxel using blood-based biomarkers, we generated a combined risk score (CRS) combing the total number of CTCs, serum PSA and ALP levels for SD/PD prediction as CRS-TPA= 0.7414 * Total-CTC number + 0.02909 * PSA + 0.01423 * ALP, which resulted in an AUC of 0.90, p= 0.037, with a sensitivity of 84.62% and specificity of 100% when the cut-off was set at >3.45 (**Figure 2C**). The CRS-TPA performed better than serum PSA and ALP alone, although the difference was not statistically significant (p= 0.66, and 0.23 respectively), likely due to the limited sample size.

In mHSPC, epithelial- and EMTing-CTC numbers did not differentiate patients based on radiological response to docetaxel. Conversely, high mesenchymal-CTC numbers trended towards (p= 0.079) higher chance of SD/PD (**Figure 2B**) with an AUC of 0.65, p= 0.099 (**Figure 2D**). Serum ALP levels were best able to predict SD/PD, with an AUC of 0.68, p= 0.077, compared to that of serum PSA which had an AUC of 0.52, p= 0.82 (**Figure 2D**). A CRS comprised of mesenchymal-CTC number and serum ALP levels as CRS-MA= 0.3599 * mesenchymal-CTC number + 0.002037 * ALP, increased the AUC to 0.69, however with only a trend towards significance (p= 0.064) (**Figure 2D**).

### CTCs were significantly associated with PFS and OS in mCRPC and mHSPC patients

**Figure 3:**
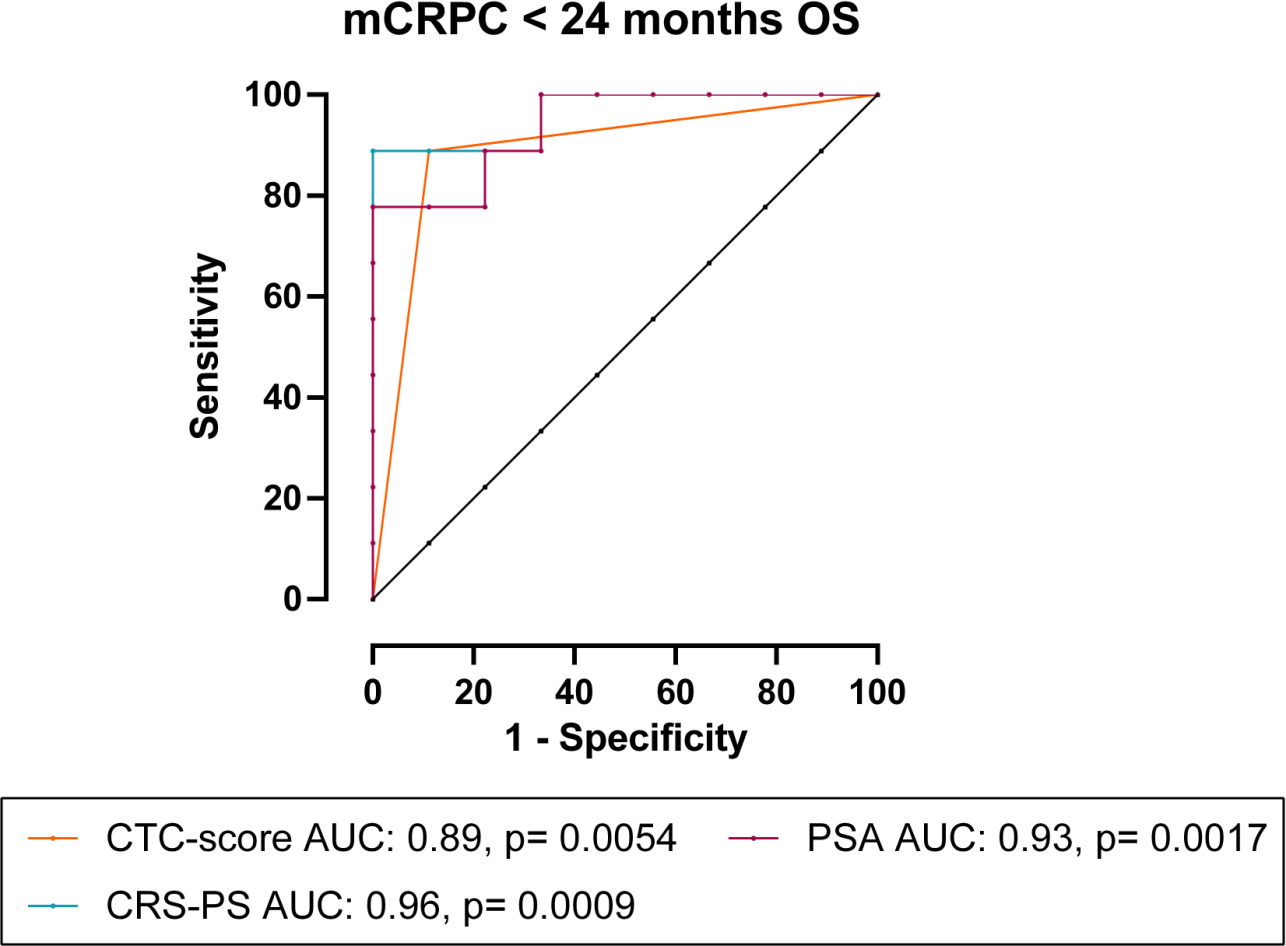
CRS of CTC-score and PSA for the prediction of <24 months OS in mCRPC patients. In mCRPC patients, combining CTC-score with PSA as CRS-PS= 0.08127 * PSA + 4.159 * CTC-score, increased the AUC to 0.96, p= 0.0009.

To assess the prognostic value of CTCs, we correlated CTC measurements with patient OS and PFS. Long term follow-up data was available for 18 mCRPC and 42 mHSPC patients. The median follow-up time for mCRPC patients was 22.7 months (range 8.0-53.1 months), during which time 13/18 (72%) patients progressed and/or died. Spearman’s correlation (**Table 3**) showed that OS significantly inversely correlated with total- (ρ= -0.66, p= 0.0027), epithelial- (ρ= -0.62, p= 0.0057) and EMTing-CTC (ρ= -0.65, p= 0.0034) numbers and a positive CTC-score (ρ= -0.80, p< 0.0001) in the mCRPC cohort. Additionally, PFS was significantly inversely correlated with epithelial-CTC numbers (ρ= -0.63, p= 0.0049) and a positive CTC-score (ρ= -0.65, p= 0.0033). We also evaluated the performance of serum biomarkers PSA and ALP. We found that serum PSA levels significantly inversely correlated with OS (ρ= -0.72, p= 0.0008) and PFS (ρ= -0.50, p= 0.035). With a view to improve the sensitivity and specificity of pre-docetaxel biomarkers for the prediction of OS, we generated a combined risk score using both PSA and CTC-score data. The AUC of a CRS comprised of PSA (AUC= 0.93) and CTC-score (AUC= 0.89) (CRS-PS= 0.08127 * PSA + 4.159 * CTC-score) to discriminate mCRPC patients with <24 months OS from those with ≥24 months OS reached 0.96, p= 0.0009, with a sensitivity of 88.9% and a specificity of 100% when the cut-off was set to <5.96 (**Figure 3**). This made an improvement on the AUC of PSA alone but without significance, p= 0.60.

**Table 3:** Spearman’s correlation of CTCs and serum biomarkers with OS and PFS.

	OS	PFS
	*Spearman’s ρ (p-value)*	
mCRPC patients		
**Total-CTCs**	**-0.66 (0.0027)**	**-0.52 (0.075)**
**Epithelial-CTCs**	**-0.62 (0.0057)**	**-0.63 (0.0049)**
**EMTing-CTCs**	**-0.65 (0.0034)**	-0.14 (0.57)
**Mesenchymal-CTCs**	-0.40 (0.10)	0.34 (0.17)
**CTC-score**	**-0.80 (<0.0001)**	**-0.65 (0.0033)**
**PSA**	**-0.72 (0.0008)**	**-0.50 (0.035)**
**ALP**	-0.41 (0.13)	-0.16 (0.55)
mHSPC patients		
**Total-CTCs**	0.16 (0.31)	-0.087 (0.58)
**Epithelial-CTCs**	-0.084 (0.60)	-0.16 (0.30)
**EMTing-CTCs**	0.13 (0.41)	0.11 (0.43)
**Mesenchymal-CTCs**	0.20 (0.20)	-0.076 (0.63)
**CTC-score**	0.012 (0.94)	-0.18 (0.26)
**PSA**	-0.21 (0.19)	-0.16 (0.33)
**ALP**	**-0.45 (0.0068)**	**-0.62 (<0.0001)**

PFS, progression-free survival; OS, overall survival; bold black numbers, significant results

**Figure 4:**
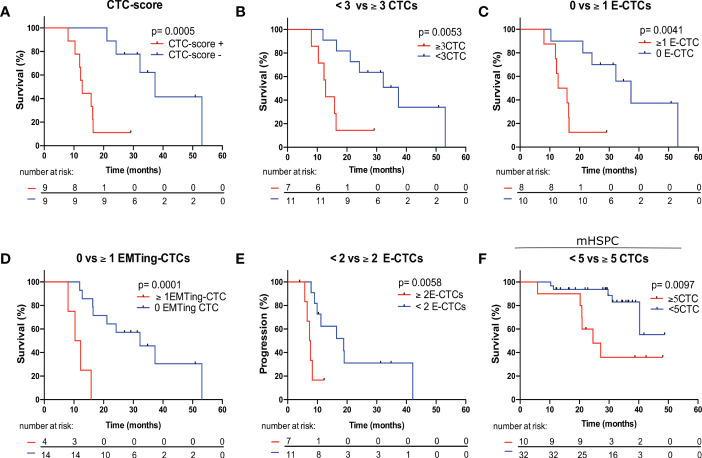
Kaplan Meier survival analysis of CTC measurements to predict OS and PFS in mCRPC and mHSPC patients. **(A)** mCRPC patients with a positive CTC-score experienced significantly shorter median OS compared to those with a negative CTC-score (12.80 vs. 37.33 months, HR= 5.08, p= 0.0005). **(B)** mCRPC patients with ≥3 total-CTCs experienced significantly shorter median OS compared to those with <3 total-CTCs (12.80 vs. 37.33 months, HR= 3.84, p= 0.0053). **(C)** mCRPC patients with ≥1 epithelial-CTCs experienced significantly shorter median OS compared to those with <1 epithelial-CTC (14.30 vs. 37.33 months, HR= 3.89, p= 0.0041). **(D)** mCRPC patients with ≥1 EMTing-CTCs experienced significantly shorter median OS compared to those with <1 EMTing-CTC (11.32 vs. 32.37 months, HR= 6.73, p= 0.0001). **(E)** mCRPC patients with ≥2 epithelial-CTCs experienced significantly shorter median PFS compared to those with <2 epithelial-CTC (7.52 vs. 18.83 months, HR= 3.93, p= 0.0058). **(F)** mHSPC patients with ≥5 CTCs experienced significantly shorter median OS compared to those with <5 CTCs (24.57 vs undefined months, HR= 4.14, p= 0.0097).

We then performed Kaplan Meier survival analysis using total and subtype CTC numbers to predict OS and PFS with optimal CTC number cut-offs evaluated (**Table 4**). In mCRPC patients, each total-CTC number cut-off that was explored (<2 vs. ≥2 to <6 vs. ≥6 CTCs) was significantly associated with patients with short median OS. The detection of a positive CTC-score (12.80 vs. 37.33 months, HR= 5.08, p= 0.0005) (**Figure 4A**), ≥3 total-CTCs (12.80 vs. 37.33 months, HR= 3.84, p= 0.0053) (**Figure 4B**), ≥1 epithelial-CTC (14.30 vs. 37.33 months, HR= 3.89, p= 0.0041) (**Figure 4C**) and ≥1 EMTing-CTC (11.32 vs. 32.37 months, HR= 6.73, p= 0.0001) (**Figure 4D**) were most significantly associated with shorter median OS. Importantly, when the Bonferroni correction method was applied to adjust p values for multiple testing, a positive CTC-score (p_adj_= 0.0055), ≥1 epithelial-CTC (p_adj_= 0.045) and ≥1 EMTing-CTC (p_adj_= 0.0011) remained significantly associated with shorter median OS. The detection of ≥2 epithelial-CTCs was most significantly associated with shorter median PFS (7.52 vs. 18.83 months, HR= 3.93, p= 0.0058) (**Figure 4E**).

**Table 4:** Kaplan Meier analysis of CTC enumeration cut-offs for OS and PFS in mCRPC and mHSPC.

CTC parameters	Patients per group (n)	OS			PFS		
		Median survival (months)	HR (95%CI)	p-value	Median survival (months)	HR (95%CI)	p-value
mCRPC patients							
CTC-score negative vs. CTC-score positive	9 vs. 9	37.33 vs. 12.80	5.08 (3.41 to 43.57)	**0.0005**	18.83 vs. 8.0	2.70 (1.18 to 15.03)	**0.042**
< 2 vs. ≥ 2 CTCs	9 vs. 9	34.80 vs. 12.80	3.13 (1.37 to 15.82)	**0.021**	16.43 vs. 8.0	1.54 (0.50 to 5.50)	0.43
< 3 vs. ≥ 3 CTCs	11 vs. 7	37.33 vs. 12.80	3.84 (2.08 to 34.72)	**0.0053**	16.43 vs. 8.0	2.03 (0.66 to 11.16)	0.20
< 4 vs. ≥ 4 CTCs	12 vs. 6	32.27 vs. 12.53	3.66 (1.80 to 39.08)	**0.010**	13.82 vs. 8.30	1.57 (0.41 to 7.95)	0.46
< 5 vs. ≥ 5 CTCs	13 vs. 5	32.27 vs. 12.27	3.39 (1.38 to 41.25)	**0.025**	11.20 vs. 9.80	1.25 (0.25 to 6.61)	0.77
< 6 vs. ≥ 6 CTCs	14 vs. 4	28.23 vs. 12.53	2.50 (0.68 to 22.90)	**0.014**	11.30 vs. 7.33	2.10 (0.38 to 21.12)	0.32
0 vs. ≥ 1 E-CTC	10 vs. 8	37.33 vs. 14.30	3.89 (2.17 to 28.45)	**0.0041**	18.83 vs. 7.70	3.57 (1.82 to 28.47)	**0.0088**
< 2 vs. ≥ 2 E-CTC	11 vs 7	37.33 vs. 12.80	3.72 (1.99 to 32.21)	**0.0065**	18.83 vs. 7.52	3.93 (2.20 to 51.88)	**0.0058**
0 vs. ≥ 1 EMTing-CTC	14 vs. 4	32.37 vs. 11.32	6.73 (11.47 to 1043)	**0.0001**	11.20 vs. 10.28	1.03 (0.23 to 4.72)	0.97
0 vs. ≥ 1 M-CTC	10 vs. 8	32.37 vs. 16.07	1.39 (0.60 to 5.29)	0.30	13.82 vs. 8.3	0.89 (0.30 to 2.61)	0.83
< 2 vs. ≥ 2 M-CTC	12 vs. 6	28.23 vs. 12.53	1.88 (0.56 to 8.89)	0.27	10.57 vs. Undefined	0.60 (0.18 to 2.31)	0.50
mHSPC patients							
CTC-score negative vs. CTC-score positive	20 vs. 22	Undefined vs. Undefined	2.53 (0.72 to 7.74)	0.15	36.40 vs. 12.52	1.90 (0.89 to 4.01)	0.10
< 2 vs. ≥ 2 CTCs	23 vs. 19	Undefined vs. 40.30	1.73 (0.53 to 5.60)	0.37	26.57 vs. 16.83	1.42 (0.67 to 3.02)	0.37
< 3 vs. ≥ 3 CTCs	27 vs. 15	Undefined vs. 40.30	2.74 (0.86 to 9.71)	**0.090**	21.80 vs. 13.33	1.58 (0.74 to 3.66)	0.23
< 4 vs. ≥ 4 CTCs	29 vs. 13	Undefined vs. 31.07	3.91 (1.31 to 16.89)	**0.018**	26.57 vs. 11.13	1.62 (0.74 to 4.06)	0.22
< 5 vs. ≥ 5 CTCs	32 vs. 10	Undefined vs. 24.57	4.14 (1.61 to 27.36)	**0.0097**	26.57 vs. 9.63	1.79 (0.77 to 5.27)	0.16
< 6 vs. ≥ 6 CTCs	34 vs. 8	Undefined vs. 24.57	3.98 (1.54 to 35.11)	**0.013**	26.57 vs. 9.63	2.18 (0.94 to 8.33)	**0.067**
0 vs. ≥ 1 E-CTC	28 vs. 14	Undefined vs. Undefined	2.86 (0.92 to 12.25)	**0.068**	29.37 vs. 11.42	2.07 (0.99 to 5.33)	**0.098**
< 2 vs. ≥ 2 E-CTC	35 vs. 7	Undefined vs. Undefined	2.07 (0.49 to 12.93)	0.27	21.80 vs. 11.13	1.54 (0.58 to 4.65)	0.39
0 vs. ≥ 1 EMTing-CTC	36 vs. 6	Undefined vs. Undefined	0.50 (0.12 to 2.81)	0.50	15.90 vs. 28.97	0.85 (0.31 to 2.34)	0.76
0 vs. ≥ 1 M-CTC	22 vs. 20	Undefined vs. 40.30	2.13 (0.65 to 7.0)	0.23	26.57 vs. 15.88	1.22 (0.57 to 2.61)	0.60
< 2 vs. ≥ 2 M-CTC	31 vs. 11	40.30 vs 31.07	1.86 (0.57 to 7.28)	0.29	26.57 vs. 13.33	1.56 (0.68 to 3.97)	0.27

PFS, progression-free survival; OS, overall survival; E-CTC, Epithelial CTC; M-CTC, Mesenchymal CTC; Undefined, The probability of survival exceeds 50% at the longest time point; bold black numbers, significant results; bold grey numbers, results with a trend towards significance.

The median follow-up time for mHSPC patients was 29.5 months (range 5.9-48.8 months), during which time 25/42 (60%) patients progressed and 11/42 (26%) died. Spearman’s correlation did not show associations of CTC measurements with PFS and OS, however, ALP was significantly inversely correlated with OS (ρ= -0.45, p= 0.0068) and PFS (ρ= -0.62, p< 0.0001) (**Table 3**). Kaplan Meier analysis revealed that patients with ≥5 CTCs experienced the most significantly shorter median OS (24.57 vs undefined months, HR= 4.14, p= 0.0097) (**Table 4**, **Figure 4F**).

### CTC gene expression predicted PFS and OS in mHSPC patients

Subsequently, we interrogated CTC mRNA expression to enhance the efficiency of CTCs as predictive biomarkers beyond CTC enumeration in the mHSPC cohort. Up-regulated differentially expressed genes in docetaxel-resistant cell lines from two microarray datasets were considered for docetaxel-resistant CTC detection. There were 162 genes commonly up-regulated in docetaxel-resistant cell lines and considered for further validation. Additionally, we identified 75 reported docetaxel-resistance related genes by literature search of relevant publications regarding the mechanisms of docetaxel resistance. These panels were combined to form a 237-candidate gene panel (**Supplementary Table 3**). As the enriched CTC fraction that is harvested from the Parsortix**®** is not pure, it was necessary to account for white blood cell contamination in the sample. Therefore, we performed two steps of gene expression analysis to exclude any of the 237 candidate genes that were expressed in leucocytes, 1. *in silico* analysis and 2. *in vitro* experiments using PCa cell lines and patient derived leucocyte samples. Firstly, the 237 genes were searched in The Genotype-Tissue Expression Portal V7 database for their expression in prostate and whole blood. Genes were selected based on their relatively high expression in prostate tissue and low/no expression in whole blood. This resulted in a candidate panel of 39 genes. Secondly, using RT-qPCR in paired docetaxel-sensitive (PC3-AG) and docetaxel-resistant (PC3-D12) PCa cell lines, and five PBMC samples from biopsy-negative patients, we experimentally validated 23/39 genes for lack of expression in leucocytes. A further seven genes, including EMT and stem cell markers (*PTPRC* (*CD45*), *SNAI1*, *ZEB1*, *NANOG*, *POU5F1*, *PROM1* and *SOX2*) were included due to their potential prognostic value in clinical samples and lack of expression in leucocytes, along with housekeeping genes *GAPDH* and *MRFAP1*. Unfortunately, the mCRPC sample size available at this time point was limited and gene expression analysis was not performed. However, the relationship between candidate gene expression and OS/PFS was investigated in 33 mHSPC patient samples.

**Figure 5:**
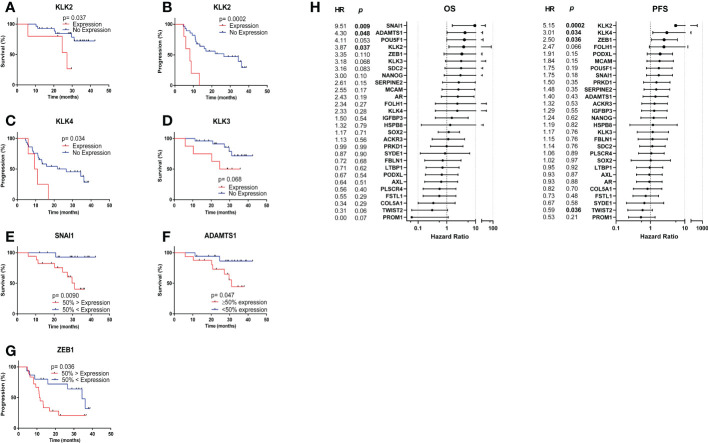
Kaplan Meier analysis based on CTC gene expression. **(A)** *KLK2* expression was significantly associated with shorter median OS (27.17 vs. undefined months, HR= 3.87, p= 0.037). **(B)** *KLK2* expression was significantly associated with shorter median PFS (8.13 vs. 26.57 months, HR= 5.15, p= 0.0002). **(C)** *KLK4* expression was significantly associated with shorter median PFS (10.17 vs. 26.57, HR= 3.01, p= 0.034). **(D)** *KLK3 (PSA)* expression had a trend towards significant association with shorter median OS (30.13 vs. undefined months, HR= 3.18, p= 0.068). **(E)** Patients with 50% highest *SNAI1* expression experienced significantly shorter median OS (31.07 vs. undefined months, HR= 9.51, p= 0.0090). **(F)** Patients with 50% highest *ADAMTS1* expression experienced significantly shorter median OS (31.07 vs undefined months, HR= 4.30, p= 0.047). **(G)** Patients with the 50% highest *ZEB1* expression experienced significantly shorter median PFS (11.50 vs. 24.40 months, HR= 2.5, p= 0.036). **(H)** Hazard ratios for each gene for OS and PFS. *Undefined months:* The probability of survival exceeds 50% at the longest time point.

Survival analysis was performed by separating patients into the following groups: 1) Expression vs. no expression 2) 50% highest expression vs. 50% lowest expression. Kaplan Meier analysis revealed that *KLK2* expression was significantly associated with shorter median OS (27.17 vs. undefined months, HR= 3.87, p= 0.037) (**Figure 5A**) and PFS (8.13 vs. 26.57 months, HR= 5.15, p= 0.0002) (**Figure 5B**). *KLK4* expression was significantly associated with shorter median PFS (10.17 vs. 26.57, HR= 3.01, p= 0.034) (**Figure 5C**), while *KLK3* (PSA) expression had only a trend towards shorter median OS (30.13 vs. undefined months, HR= 3.18, p= 0.068) (**Figure 5D**) and did not separate patients based on PFS. Patients with 50% highest *SNAI1* and/or *ADAMTS1* expression experienced significantly shorter median OS (31.07 vs. undefined months, HR= 9.51, p= 0.0090 (**Figure 5E**); 31.07 vs undefined months, HR= 4.30, p= 0.047 (**Figure 5F**), respectively) and patients with the 50% highest *ZEB1* expression experienced significantly shorter median PFS (11.50 vs. 24.40 months, HR= 2.5, p= 0.036) (**Figure 5G**). Hazard ratios for each gene for OS and PFS are presented in **Figure 5H**.

**Figure 6:**
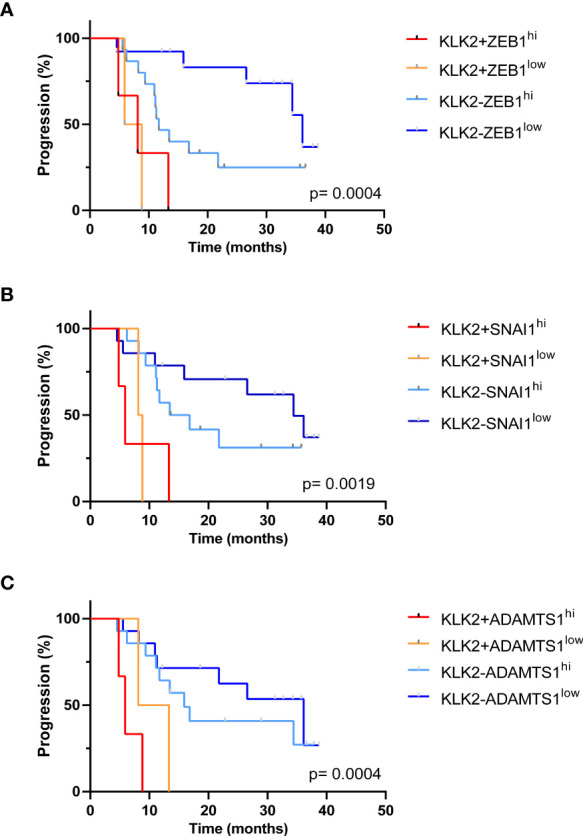
Kaplan Meier analysis of patients expressing multiple poor prognostic genes. **(A)** The median PFS for patients with *KLK2*+*ZEB1* ^hi^ was 8.13 months, which was significantly shorter than *KLK2*-*ZEB1* ^low^ group (36.13 months) with p= 0.0008. Overall Logrank p= 0.0004. **(B)** The median PFS for patients with *KLK2*+*SNAI1* ^hi^ was 5.9 months. *KLK2*+*SNAI1* ^hi^ vs. *KLK2*-*SNAI1* ^hi^: 5.9 vs. 15.15 months, p= 0.019; *KLK2*+*SNAI1* ^hi^ vs. *KLK2*-*SNAI1* ^low^: 5.9 vs. 34.40 months, p= 0.0083. Logrank p= 0.0019. **(C)** The median PFS for patients with *KLK2*+*ADAMTS1* ^hi^ was 5.9. *KLK2*+*ADAMTS1* ^hi^ vs. *KLK2*+*ADAMTS1* ^low^: 5.9 vs. 10.73 months, p= 0.28; *KLK2*+*ADAMTS1* ^hi^ vs. *KLK2*-*ADAMTS1* ^hi^: 5.9 vs 15.90 months, p= 0.0015; *KLK2*+*ADAMTS1* ^hi^ vs. *KLK2*-*ADAMTS1* ^low^: 5.9 vs. 36.13 months, p= 0.0007. Logrank p= 0.0004.

We then investigated the clinical outcomes of patients who had CTCs expressing more than one high-risk gene. The median PFS for patients with *KLK2*+*ZEB1* ^hi^ (8.13 months Logrank p= 0.0004) (**Figure 6A**) and/or *KLK2*+*SNAI1* ^hi^ (5.9 months, Logrank p= 0.0019) (**Figure 6B**) and/or *KLK2*+*ADAMTS1* ^hi^ (5.9 months, Logrank p= 0.0004) (**Figure 6C**) was significantly shorter than for patients with no *KLK2* expression and low expression of each gene. Patients expressing both genes also had high total CTC numbers detected in paired samples (*KLK2*+*ZEB1* ^hi^: ≥8 CTCs, *KLK2*+*SNAI1* ^hi^: ≥5 CTCs, *KLK2*+*ADAMTS1* ^hi^: 0, 5 and 9 CTCs).

**Figure 7:**
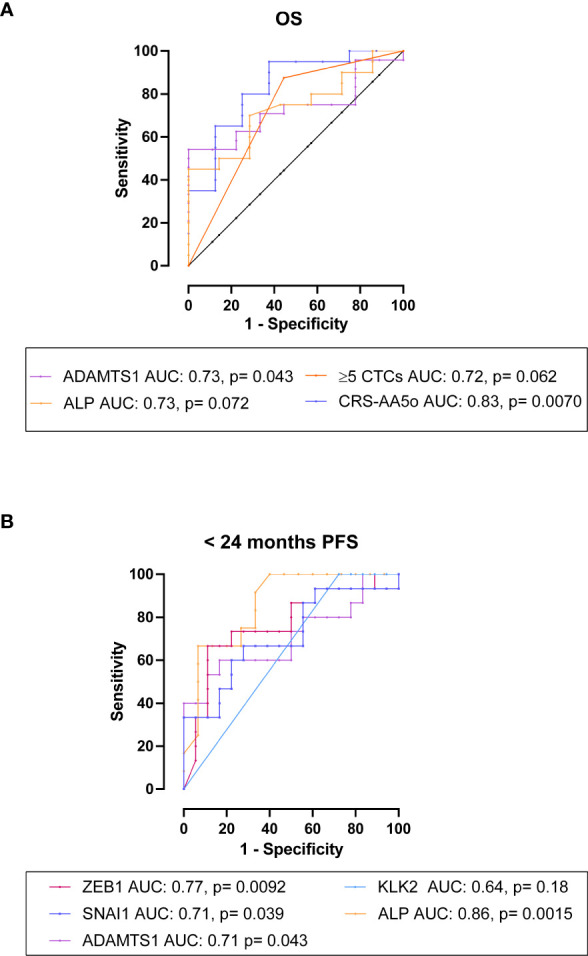
The association and predictive value of CTC gene expression with OS and PFS in mHSPC patients. **(A)** ROC analysis for OS: High expression of *ADAMTS1* had an AUC of 0.73, p= 0.043 for the prediction of OS. Neither serum PSA or ALP levels significantly differentiated patients based on OS, although ALP showed a trend towards significance (AUC= 0.55, p= 0.66; AUC= 0.73, p= 0.072, respectively). Combining *ADAMTS1*, ALP and ≥5 CTCs as CRS-AA5o= 0.06386*ADAMTS1 + 0.001465*ALP + 2.169*≥5CTCs, increased the AUC to 0.83, p= 0.0070. **(B)** ROC analysis for <24 months PFS: High expression of *ZEB1*, *SNAI1* and *ADAMTS1* was significantly predictive of <24 months PFS (AUC= 0.77, p= 0.0092; AUC= 0.71, p= 0.039; AUC= 0.71, p= 0.043, respectively). Additionally, ALP but not serum PSA levels showed significant AUCs (AUC= 0.86, p= 0.0015; AUC= 0.52, p= 0.86, respectively).

Using receiver operating characteristic curve analysis, high expression of *ADAMTS1* was significantly predictive of shorter OS with an AUC of 0.73, p= 0.043 (**Figure 7A**). Neither serum PSA or ALP levels significantly differentiated patients based on OS, although ALP showed a trend towards significance (AUC= 0.55, p= 0.66; AUC= 0.73, p= 0.072, respectively) (**Figure 7A**). Combining *ADAMTS1*, ALP and ≥5 total CTCs to form a CRS as CRS-AA5o= 0.06386 * *ADAMTS1* + 0.001465 * ALP + 2.169 * ≥5 total CTCs, which increased the AUC to 0.83, p= 0.0070 (65% sensitivity and 87.5% specificity at a cut off of <0.48) vs AUC of 0.73 for ALP alone, (**Figure 7A**) however without a significant difference (p= 0.38).

We then evaluated the candidate genes for the prediction of PFS and found that high expression of *ZEB1, SNAI1* and *ADAMTS1* was significantly predictive of progression within 24 months (AUC= 0.77, p= 0.0092; AUC= 0.71, p= 0.039; AUC= 0.71, p= 0.043, respectively) (**Figure 7B**). Expression of *KLK2* had a trend of correlated with <24 months PFS, but the AUC was not significant (AUC= 0.64, p= 0.18) (**Figure 7B**). ALP but not serum PSA levels significantly discriminated patients with <24 months PFS from those with ≥24 months PFS (AUC= 0.86, p= 0.0015; AUC= 0.52, p= 0.86, respectively) (**Figure 7B**).

## Discussion

Improvements in our understanding of the genetic landscape of PCa have advanced treatments for metastatic disease. These treatments comprise androgen-receptor targeting therapies (abiratorine, enzalutminde) bone-targeting radiotherapies (Radium-223), immunotherapies, and cytotoxic chemotherapies (docetaxel, cabazataxel). Docetaxel is now a first-line therapy in both mCRPC and mHSPC, however its efficacy is limited by the vast inter- and intra-tumour heterogeneity of PCa, resulting in clonal populations with inherent and/or acquired resistance in a proportion of patients. Although multiple docetaxel resistance mechanisms have been revealed through extensive research, such as upregulation of drug efflux pumps (e.g. ABCB1) (40), alterations to β-tubulin and expression of tubulin isoforms (41), deregulation of apoptosis and survival signalling pathways (42), induction of EMT and cancer stem cells phenotypes (36, 43, 44), and deregulation of AR signalling (45), the development of clinically useful tools to predict response is currently still required (46–48). Thus, patients with resistance may undergo systemic chemotherapy with little survival benefit (49, 50). Our ability to elucidate biomarkers of resistance is limited by tissue biopsy, which samples only a small fraction of the entire heterogeneous tumour, is practically difficult in metastatic disease and as a repeated measure. However, liquid biopsy analysis of CTCs offers a minimally invasive, easily repeatable tool for cancer specific interrogation. Using the Parsortix^®^ CTC isolation system, we investigated the potential of using CTCs, including subtype and CTC gene expression analysis, to predict docetaxel response and survival benefit in both mCRPC and mHSPC patients.

We found that individual CTC measurements alone showed some, but limited associations with RECIST response in mCRPC and mHSPC patients. However, when combined with serum biomarkers in mCRPC patients, initial progressive disease could be predicted with high accuracy. Furthermore, combining CTCs with serum biomarkers efficiently detected mCRPC patients at risk of shorter OS, supporting the potential use of CTCs to triage patients for docetaxel treatment. Importantly, we showed that ≥5 pre-docetaxel CTCs/7.5mL were also significantly associated with poor PFS in mHSPC patients treated with first-line chemo-hormonal therapy. Additionally, molecular analysis of CTC samples revealed that the expression of the candidate docetaxel-resistance gene *ADAMTS1*, and EMT transcription factors *ZEB1* and *SNAI1* along with the PCa specific kallikreins *KLK2* and *KLK4* significantly correlated with poor mHSPC patient outcome.

While the detection of CTCs is now a well-established marker of aggressive cancer with poor survival outcome, it is yet to be determined if CTCs have an association with initial docetaxel response. Immediate radiological or RECIST response criteria are commonly used to determine a treatment response upon the completion of a therapeutic regimen. In our study, although potentially due to the limited cohort sizes, individual CTC subtypes did not significantly discriminate between patients with a partial response and those with stable disease and/or progressive disease. This may suggest that in the pre-docetaxel setting the CTC subtype has limited relation to inherent docetaxel sensitivity and RECIST response. Nevertheless, we found that total CTC numbers showed potential for predicting initial radiological treatment response when used in combination with serum biomarkers (PSA and ALP) in mCRPC patients, with a good AUC of 0.90. Although we observed a higher number of mesenchymal CTC in mHSPC patients lacking a partial response to treatment, they did not add significant predictive value. Newly diagnosed mHSPC patients are treated with first-line ADT for a short period prior to starting and throughout docetaxel therapy. Unlike mCRPC patients, all mHSPC patients should be responsive to ADT at this time. Therefore, our results indicate that responsiveness to first-line ADT might affect the value of CTCs for the prediction of near-term clinical response to docetaxel. This is also reflected in the lack of significant correlations between serum PSA level and CTC numbers before docetaxel treatment in mHSPC patients. In summary, CTCs might have a potential value in predicting docetaxel response, but data here is insufficient to make a conclusion. Further investigations in larger cohorts are required.

While pre-docetaxel CTC measurements showed limitations in the prediction of initial radiological response, we showed that CTCs were associated with OS and PFS subsequent to docetaxel treatment. The significant correlation of epithelial CTCs with shorter OS and/or PFS confirmed previous research findings in mCRPC patients to be treated with docetaxel (19, 23, 24, 26, 27, 51). However, by using the epitope independent Partsortix^®^ isolation system, we were able to capture and analyse three different CTC subtypes. Interestingly, in the mCRPC patient cohort, the detection of ≥1 EMTing-CTCs (p= 0.0001) and a positive CTC-score (p= 0.0005) were the most significant predictors of shorter OS among the different CTC measurements. Our previous studies have demonstrated both to be valuable PCa biomarkers, and associated with increased metastatic burden (32, 38). Patients with a positive-CTC score and/or ≥1 EMTing-CTC present in 7.5mL of pre-docetaxel blood experienced an approximate three-fold reduction in median OS time compared to those with a negative CTC-score or no EMTing-CTCs. This demonstrates the value of analysing different subtypes of CTCs and the good prognostic value of the CTC score, in which mesenchymal CTCs were a component. A positive CTC-score combined with serum PSA (AUC= 0.96 for OS <24 months) may flag high-risk mCRPC patients with high sensitivity (88.89%) and specificity (100%) and facilitate timely therapeutic intervention post-docetaxel, such as Cabazitaxel administration, which has been shown to retain activity in patients after docetaxel treatment (52).

To date, reports on the use of CTCs as a prognostic biomarker in mHSPC patients treated with ADT plus docetaxel are limited. The prediction of significantly shorter OS by ≥5 total-CTCs in mHSPC patients may be useful for treatment stratification in this cohort. In this study, CTCs expressing cytokeratin alone did not significantly differentiate mHSPC patients with shorter OS from those with a good response and prolonged OS after docetaxel treatment. Again, these findings highlight the advantage of epitope independent CTC isolation, which allows for the capture and analysis of multiple CTC subtypes.

We showed that the RNA expression of certain genes in CTCs correlated with shorter OS and/or PFS in mHSPC patients. These poor prognostic genes included the EMT transcription factors *ZEB1* and *SNAI1*. EMT is responsible for tumour cell migration and metastasis, and is associated with drug resistance in multiple solid tumour types (53). In docetaxel resistance, upregulation of EMT genes has been shown to mediate resistance emergence in PCa cell line models (36, 54, 55) and increased expression in primary tumours prior to therapy has been correlated with radiological relapse (36). Several EMT genes have been assessed in CTCs, the most common being Vimentin, for detection of a mesenchymal subtypes which have been linked to higher metastatic burden, a more aggressive phenotype and disease progression in PCa (32, 56, 57). The shorter OS and/or PFS associated with the RNA expression of *ZEB1* and *SNAI1* by CTCs may indicate a more aggressive and potentially docetaxel resistant phenotype being present in the primary or metastatic tumour sites, which may promote disease progression in mHSPC patients. Additionally, these findings highlight the importance of considering multiple different markers corresponding to the same cellular phenotype, as genes may be exhibiting different patterns of spatial–temporal expression under pathological conditions, yet could be controlled by different upstream signals.

Patients with mHSPC who had detectable *KLK2* RNA expression in CTCs were more likely to suffer shorter PFS compared to patients with *KLK3* (PSA) expression. The protein encoded by *KLK2*, hK2, has been utilised in the 4Kscore^®^ Test to predict risk of aggressive PCa (58). Our findings suggest that *KLK2*/hK2 may also be used as biomarker to identify mHSPC patients who are likely to progress under chemo-hormonal treatment.

The metalloprotease, *ADAMTS1*, was upregulated in docetaxel-resistant cells in microarray analysis and was associated with both PFS and OS. Combining *ADAMTS1* expression with ALP levels and ≥5 CTCs predicted shorter median OS with high sensitivity and specificity. For PFS, ALP was highly predictive, with an AUC of 0.86, leaving little margin for improvement, so combined biomarker analysis was not performed.

The variety of CTC derived genes that were identified as biomarkers of poor prognosis indicates the heterogeneity of CTCs between patients. CTC heterogeneity may be the result of a spatio-temporally different microenvironment surrounding the tumour lesions, in the circulation, as well as differences in therapy response (14, 59). In the case of the mHSPC patient cohort, differing levels of response to initial ADT may alter tumour biology, influence CTC gene expression and fitness, and subsequent response to docetaxel therapy, such as induction of EMT machinery (60–62). To explore this, further analysis of CTC gene expression changes over multiple time points during therapy in warranted.

The limitations of this study include: 1. Small patient cohort, particularly for the mCRPC patients, although we observed several significant corrrelations. The small sample size was due to the several effective therapies been devloped in recently years for PCa, leading to competing treatment options. 2. The candidate CTC genes were selected based on the microarray dataset gene expression profile from 2D-cultured docetaxel-resistance cell line models, as currently, datasets from better models for docetaxel resistant versus sensitive samples are not available. It is well-known that 2D-induced resistance creates artificial resistance mechanisms. Therefore, validation of these candidate genes in clinically relevant samples is critical. 3. As the harvested CTC samples that we used for gene expression analysis were not pure CTCs (with predominantly leucocyte contamination), we had to exclude a large number of candidate docetaxel resistance genes which were expressed in leucocyte. This led to only 23 selected from the initial 237 candidate genes, thus potentially missing genes with good docetaxel therapeutic response prediction value. Further pure CTC selection (although a challenging task) or single cell RNA sequencing may be explored in the future to address this issue.

In summay, our study demonstrated that in mCRPC, elevated numbers of CTCs were inidcators of poor initial response when combined with serum biomarkers, and CTC measurements could be used to predict short OS and/or PFS in mCRPC and mHSPC patients. Addtionally, we showed that measuring RNA expression of candidate docetaxel-resistance and PCa related genes from CTC samples increased our ability to predict patient outcome in the mHSPC patient cohort. Importantly, we found that combining CTC data with clinical serum biomarkers has the potential to predict poor docetaxel treatment response, although this should be confirmed in a large series of samples.

## Data availability statement

The datasets presented in this study can be found in online repositories. The names of the repository/repositories and accession number(s) can be found in the article/**Supplementary Material**.

## Ethics statement

The studies involving human participants were reviewed and approved by UK Research Ethics Committee: London - City & East Research Ethics Committee. The patients/participants provided their written informed consent to participate in this study.

## Author contributions

Conceptualization: CD, PR, AG, GSh, JS, and Y-JL. Validation: CD and LX. Formal analysis: CD and Y-JL. Investigation: CD, TG, EB, ES, LX, and XM. Data Curation: CD, EB, ES, XM, and GSc. Writing – Original Draft: CD. Visualization: CD. Writing – Review and Editing: TG, ES, XM, PR, AG, TO, JS, and Y-JL. Methodology: XM, Y-JL, LX. Resources: GSh, PR, KT, CA, AW, MG, SC, TP, AG, SK, GSc, DB, and JS. Supervision: JS and Y-JL. Project administration: Y-JL. Funding: Y-JL. All authors contributed to the article and approved the submitted version.

## References

[1] SEER. Cancer stat facts: Prostate cancer. (2018). Available at: https://seer.cancer.gov/statfacts/html/prost.html

[2] Chandrasekar T. Mechanisms of resistance in castration-resistant prostate cancer (CRPC). Transl Androl Urol (2015) 4(3):365–80. doi: 10.3978/j.issn.2223-4683.2015.05.02 PMC4708226 26814148

[3] Tannock IF de Wit R Berry WR Horti J Pluzanska A Chi KN. Docetaxel plus prednisone or mitoxantrone plus prednisone for advanced prostate cancer. N Engl J Med (2004) 351(15):1502–12. doi: 10.1056/NEJMoa040720 15470213

[4] Sweeney CJ Chen Y Carducci M Liu G Jarrard DF Eisenberger M. Chemohormonal therapy in metastatic hormone-sensitive prostate cancer. N Engl J Med (2015) 373(8):737–46. doi: 10.1056/NEJMoa1503747 PMC4562797 26244877

[5] James ND Sydes MR Clarke NW Mason MD Dearnaley DP Spears MR. Addition of docetaxel, zoledronic acid, or both to first-line long-term hormone therapy in prostate cancer (STAMPEDE): survival results from an adaptive, multiarm, multistage, platform randomised controlled trial. Lancet (2016) 387(10024):1163–77. doi: 10.1016/S0140-6736(15)01037-5 PMC4800035 26719232

[6] Kyriakopoulos CE Chen Y Carducci MA Liu G Jarrard DF Hahn NM. Chemohormonal therapy in metastatic hormone-sensitive prostate cancer: Long-term survival analysis of the randomized phase III E3805 CHAARTED trial. J Clin Oncol (2018) 36(11):1080–7. doi: 10.1200/JCO.2017.75.3657 PMC5891129 29384722

[7] Varnai R Koskinen LM Mäntylä LE Szabo I FitzGerald LM Sipeky C. Pharmacogenomic biomarkers in docetaxel treatment of prostate cancer: From discovery to implementation. Genes (2019) 10(8):0599. doi: 10.3390/genes10080599 PMC6723793 31398933

[8] Zhao L Lee BY Brown DA Molloy MP Marx GM Pavlakis N. Identification of candidate biomarkers of therapeutic response to docetaxel by proteomic profiling. Cancer Res (2009) 69(19):7696–703. doi: 10.1158/0008-5472.CAN-08-4901 19773444

[9] Sekino Y Teishima J. Molecular mechanisms of docetaxel resistance in prostate cancer. Cancer Drug Resist (2020) 3(4):676–85. doi: 10.20517/cdr.2020.37 PMC8992564 35582222

[10] O'Neill AJ Prencipe M Dowling C Fan Y Mulrane L Gallagher WM. Characterisation and manipulation of docetaxel resistant prostate cancer cell lines. Mol Cancer (2011) 10(1):126. doi: 10.1186/1476-4598-10-126 21982118 PMC3203088

[11] Marín-Aguilera M Codony-Servat J Kalko SG Fernández PL Bermudo R Buxo E. Identification of docetaxel resistance genes in castration-resistant prostate cancer. Mol Cancer Ther (2012) 11(2):329–39. doi: 10.1158/1535-7163.MCT-11-0289 22027694

[12] Ben-Hamo R Berger AJ Gavert N Miller M Pines G Oren R. Predicting and affecting response to cancer therapy based on pathway-level biomarkers. Nat Commun (2020) 11(1):3296. doi: 10.1038/s41467-020-17090-y 32620799 PMC7335104

[13] Scher HI Morris MJ Larson S Heller G. Validation and clinical utility of prostate cancer biomarkers. Nat Rev Clin Oncol (2013) 10(4):225–34. doi: 10.1038/nrclinonc.2013.30 PMC3790270 23459624

[14] Lin D Shen L Luo M Zhang K Li J Yang Q. Circulating tumor cells: biology and clinical significance. Signal Transd Target Ther (2021) 6(1):404. doi: 10.1038/s41392-021-00817-8 PMC8606574 34803167

[15] Micalizzi DS Maheswaran S Haber DA. A conduit to metastasis: Circulating tumor cell biology. Genes Dev (2017) 31(18):1827–40. doi: 10.1101/gad.305805.117 PMC5695084 29051388

[16] Lohr JG Adalsteinsson VA Cibulskis K Choudhury AD Rosenberg M Cruz-Gordillo P. Whole-exome sequencing of circulating tumor cells provides a window into metastatic prostate cancer. Nat Biotechnol (2014) 32(5):479–84. doi: 10.1038/nbt.2892 PMC4034575 24752078

[17] Goodman OB Symanowski JT Loudyi A Fink LM Ward DC Vogelzang NJ. Circulating tumor cells as a predictive biomarker in patients with hormone-sensitive prostate cancer. Clin Genitour Cancer (2011) 9(1):31–8. doi: 10.1016/j.clgc.2011.04.001 21705286

[18] León-Mateos L Casas H Abalo AVieito M Abreu M Anido U. Improving circulating tumor cells enumeration and characterization to predict outcome in first line chemotherapy mCRPC patients. Oncotarget (2017) 8(33):54708–21. doi: 10.18632/oncotarget.18025 PMC5589615 28903376

[19] de Bono JS Scher HI Montgomery RB Parker C Miller MC Tissing H. Circulating tumor cells predict survival benefit from treatment in metastatic castration-resistant prostate cancer. Clin Cancer Res (2008) 14(19):6302–9. doi: 10.1158/1078-0432.CCR-08-0872 18829513

[20] Lorente D Olmos D Mateo J Bianchini D Seed G Fleisher M. Decline in circulating tumor cell count and treatment outcome in advanced prostate cancer. Eur Urol (2016) 70(6):985–92. doi: 10.1016/j.eururo.2016.05.023 PMC5568108 27289566

[21] Heller G McCormack R Kheoh T Molina A Smith MR Dreicer R. Circulating tumor cell number as a response measure of prolonged survival for metastatic castration-resistant prostate cancer: A comparison with prostate-specific antigen across five randomized phase III clinical trials. J Clin Oncol (2018) 36(6):572–80. doi: 10.1200/JCO.2017.75.2998 PMC5815402 29272162

[22] Pantel K Hille C Scher HI. Circulating tumor cells in prostate cancer: From discovery to clinical utility. Clin Chem (2019) 65(1):87–99. doi: 10.1373/clinchem.2018.287102 30602476

[23] Scher HI Jia X de Bono JS Fleisher M Pienta KJ Raghavan D. Circulating tumour cells as prognostic markers in progressive, castration-resistant prostate cancer: a reanalysis of IMMC38 trial data. Lancet Oncol (2009) 10(3):233–9. doi: 10.1016/S1470-2045(08)70340-1 PMC2774131 19213602

[24] Resel Folkersma L Gómez CO Manso LSJ de Castro SV Romo IG Lázaro MV. Immunomagnetic quantification of circulating tumoral cells in patients with prostate cancer: clinical and pathological correlation. Arch Esp Urol (2010) 63(1):23–31. 20157216

[25] Scher HI Morris MJ Kelly WK Schwartz LH Heller G. Prostate cancer clinical trial end points: "RECIST"ing a step backwards. Clin Cancer Res (2005) 11(14):5223–32. doi: 10.1158/1078-0432.CCR-05-0109 PMC1852496 16033840

[26] Vogelzang NJ Fizazi K Burke JM De Wit R Bellmunt J Hutson TE. Circulating tumor cells in a phase 3 study of docetaxel and prednisone with or without lenalidomide in metastatic castration-resistant prostate cancer. Eur Urol (2017) 71(2):168–71. doi: 10.1016/j.eururo.2016.07.051 27522164

[27] Petrylak DP Vogelzang NJ Budnik N Wiechno PJ Sternberg CN Doner K. Docetaxel and prednisone with or without lenalidomide in chemotherapy-naive patients with metastatic castration-resistant prostate cancer (MAINSAIL): A randomised, double-blind, placebo-controlled phase 3 trial. Lancet Oncol (2015) 16(4):417–25. doi: 10.1016/S1470-2045(15)70025-2 25743937

[28] Armstrong AJ Luo J Nanus DM Giannakakou P Szmulewitz RZ Danila DC. Prospective multicenter study of circulating tumor cell AR-V7 and taxane versus hormonal treatment outcomes in metastatic castration-resistant prostate cancer. JCO Precis Oncol (2020) 4):1285–301. doi: 10.1200/PO.20.00200 PMC7608579 33154984

[29] Goldkorn A Plets M Agarwal N Hussain M Lara P Vaena DA. Circulating tumor cells (CTCs) in SWOG S1216: A phase 3 multicenter trial in metastatic hormone sensitive prostate cancer (mHSPC). J Clin Oncol (2016) 34(15_suppl):11516–6. doi: 10.1200/JCO.2016.34.15_suppl.11516

[30] Reichert ZR Kasputis T Nallandhighal S Abusamra SM Kasputis A Haruray S. Multigene profiling of circulating tumor cells (CTCs) for prognostic assessment in treatment-na&iuml;ve metastatic hormone-sensitive prostate cancer (mHSPC). Int J Mol Sci (2022) 23(1):4. doi: 10.3390/ijms23010004 PMC8744626 35008431

[31] Josefsson A Damber J-E Welén K. AR-V7 expression in circulating tumor cells as a potential prognostic marker in metastatic hormone-sensitive prostate cancer. Acta Oncol (2019) 58(11):1660–4. doi: 10.1080/0284186X.2019.1637540 31286815

[32] Xu L Mao X Guo T Chan PY Shaw G Hines J. The novel association of circulating tumor cells and circulating megakaryocytes with prostate cancer prognosis. Clin Cancer Res (2017) 23(17):5112–22. doi: 10.1158/1078-0432.CCR-16-3081 28615267

[33] Garg M. Epithelial, mesenchymal and hybrid epithelial/mesenchymal phenotypes and their clinical relevance in cancer metastasis. Expert Rev Mol Med (2017) 19:e3. doi: 10.1017/erm.2017.6 28322181

[34] Markiewicz A Topa J Nagel A Skokowski J Seroczynska B Stokowy T. Spectrum of epithelial-mesenchymal transition phenotypes in circulating tumour cells from early breast cancer patients. Cancers (2019) 11(1):0059. doi: 10.3390/cancers11010059 PMC6356662 30634453

[35] Dudas J. Epithelial to mesenchymal transition: A mechanism that fuels cancer Radio/Chemoresistance. Cells (2020) 9(2):0428. doi: 10.3390/cells9020428 PMC7072371 32059478

[36] Marín-Aguilera M Codony-Servat J Reig O Lozano JJ Fernandez PL Pereira MV. Epithelial-to-mesenchymal transition mediates docetaxel resistance and high risk of relapse in prostate cancer. Mol Cancer Ther (2014) 13(5):1270–84. doi: 10.1158/1535-7163.MCT-13-0775 24659820

[37] Xu L Mao X Imrali A Syed F Mutsvangwa K Berney D. Optimization and evaluation of a novel size based circulating tumor cell isolation system. PloS One (2015) 10(9):e0138032. doi: 10.1371/journal.pone.0138032 26397728 PMC4580600

[38] Xu L Mao X Grey A Scandura G Guo T Burke E. Noninvasive detection of clinically significant prostate cancer using circulating tumor cells. J Urol (2020) 203(1):73–82. doi: 10.1097/JU.0000000000000475 31389764

[39] Domingo-Domenech J Vidal SJ Rodriguez-Bravo V Castillo-Martin M Quinn SA Rodriguez-Barrueco R. Suppression of acquired docetaxel resistance in prostate cancer through depletion of notch- and hedgehog-dependent tumor-initiating cells. Cancer Cell (2012) 22(3):373–88. doi: 10.1016/j.ccr.2012.07.016 PMC5989708 22975379

[40] Robey RW Pluchino KM Hall MD Fojo AT Bates SE Gottesman MM. Revisiting the role of ABC transporters in multidrug-resistant cancer. Nat Rev Cancer (2018) 18(7):452–64. doi: 10.1038/s41568-018-0005-8 PMC6622180 29643473

[41] Hara T Ushio K Nishiwaki M Kouno J Araki H Hikichi Y. A mutation in beta-tubulin and a sustained dependence on androgen receptor signalling in a newly established docetaxel-resistant prostate cancer cell line. Cell Biol Int (2010) 34(2):177–84. doi: 10.1042/CBI20090030 19947927

[42] Pommier Y Sordet O Antony S Hayward RL Kohn KW. Apoptosis defects and chemotherapy resistance: molecular interaction maps and networks. Oncogene (2004) 23(16):2934–49. doi: 10.1038/sj.onc.1207515 15077155

[43] Skvortsov S Skvortsova I Tang DG Dubrovska A. Concise review: Prostate cancer stem cells: Current understanding. Stem Cells (2018) 36(10):1457–74. doi: 10.1002/stem.2859 PMC7903656 29845679

[44] Lai C-J Lin C Liao W Hour T Wang H Chuu C. CD44 promotes migration and invasion of docetaxel-resistant prostate cancer cells likely *via* induction of hippo-yap signaling. Cells (2019) 8(4):295. doi: 10.3390/cells8040295 30935014 PMC6523775

[45] Liu L Lou N Li X Xu G Ruan H Xiao W. Calpain and AR-V7: Two potential therapeutic targets to overcome acquired docetaxel resistance in castration-resistant prostate cancer cells. Oncol Rep (2017) 37(6):3651–9. doi: 10.3892/or.2017.5623 28498452

[46] Sandler HM Karrison T Sartor AO Gomella LG Amin MB Purdy J. Adjuvant docetaxel for high-risk localized prostate cancer: Update of NRG Oncology/RTOG 0521. J Clin Oncol (2020) 38(6_suppl):333–3. doi: 10.1200/JCO.2020.38.6_suppl.333

[47] Brady L Kriner M Coleman I Morrissey C Roudier M True LD. Inter- and intra-tumor heterogeneity of metastatic prostate cancer determined by digital spatial gene expression profiling. Nat Commun (2021) 12(1):1426. doi: 10.1038/s41467-021-21615-4 33658518 PMC7930198

[48] Lima TS Iglesias-Gato D Souza LDO Stenvang J Lima DS Røder MA. Molecular profiling of docetaxel-resistant prostate cancer cells identifies multiple mechanisms of therapeutic resistance. Cancers (2021) 13(6):1290. doi: 10.3390/cancers13061290 33799432 PMC7998254

[49] Wilson TR Johnston PG Longley DB. Anti-apoptotic mechanisms of drug resistance in cancer. Curr Cancer Drug Targets (2009) 9(3):307–19. doi: 10.2174/156800909788166547 19442051

[50] Zahreddine H Borden KL. Mechanisms and insights into drug resistance in cancer. Front Pharmacol (2013) 4:28. doi: 10.3389/fphar.2013.00028 23504227 PMC3596793

[51] Okegawa T Itaya N Hara H Tambo M Nutahara K. Circulating tumor cells as a biomarker predictive of sensitivity to docetaxel chemotherapy in patients with castration-resistant prostate cancer. Anticancer Res (2014) 34(11):6705–10. 25368278

[52] Oudard S. TROPIC: Phase III trial of cabazitaxel for the treatment of metastatic castration-resistant prostate cancer. Future Oncol (2011) 7(4):497–506. doi: 10.2217/fon.11.23 21463139

[53] De Las Rivas J Brozovic A Izraelz S Casas-Pais A Witz IP Figuroa A. Cancer drug resistance induced by EMT: novel therapeutic strategies. Arch Toxicol (2021) 95(7):2279–97. doi: 10.1007/s00204-021-03063-7 PMC8241801 34003341

[54] Hanrahan K O'Neill A Prencipe M Bugler J Murphy L Fabre A. The role of epithelial–mesenchymal transition drivers ZEB1 and ZEB2 in mediating docetaxel-resistant prostate cancer. Mol Oncol (2017) 11(3):251–65. doi: 10.1002/1878-0261.12030 PMC5527446 28133913

[55] Zhang G Tian X Li Y Wang Z Li X Zhu C. miR-27b and miR-34a enhance docetaxel sensitivity of prostate cancer cells through inhibiting epithelial-to-mesenchymal transition by targeting ZEB1. Biomed Pharmacother (2018) 97:736–44. doi: 10.1016/j.biopha.2017.10.163 29102917

[56] Mitra A Mishra L Li S. EMT, CTCs and CSCs in tumor relapse and drug-resistance. Oncotarget (2015) 6(13):10697–711. doi: 10.18632/oncotarget.4037 PMC4484413 25986923

[57] Satelli A Batth I Brownlee Z Mitra A Zhou S Noh H. EMT circulating tumor cells detected by cell-surface vimentin are associated with prostate cancer progression. Oncotarget (2017) 8(30):49329–37. doi: 10.18632/oncotarget.17632 PMC5564771 28521303

[58] Punnen S Pavan N Parekh DJ. Finding the wolf in sheep's clothing: The 4Kscore is a novel blood test that can accurately identify the risk of aggressive prostate cancer. Rev Urol (2015) 17(1):3–13. 26028995 PMC4444768

[59] Swanton C. Intratumor heterogeneity: evolution through space and time. Cancer Res (2012) 72(19):4875–82. doi: 10.1158/0008-5472.CAN-12-2217 PMC3712191 23002210

[60] Khan T Scott KF Becker TM Lock J Nimir M Ma Y. The prospect of identifying resistance mechanisms for castrate-resistant prostate cancer using circulating tumor cells: Is epithelial-to-Mesenchymal transition a key player? Pros Cancer 2020 (2020), 7938280. doi: 10.1155/2020/7938280 PMC7149487 32292603

[61] Tsai Y-C Chen W Abou-Kheir W Zeng T Yin JJ Bahmad H. Androgen deprivation therapy-induced epithelial-mesenchymal transition of prostate cancer through downregulating SPDEF and activating CCL2. Biochim Biophys Acta (BBA) - Mol Basis Dis (2018) 1864(5 Part A):1717–27. doi: 10.1016/j.bbadis.2018.02.016 29477409

[62] Karantanos T Corn PG Thompson TC. Prostate cancer progression after androgen deprivation therapy: Mechanisms of castrate resistance and novel therapeutic approaches. Oncogene (2013) 32(49):5501–11. doi: 10.1038/onc.2013.206 PMC3908870 23752182

