# Supplementary material for: The potential of using circulating tumour cells and their gene expression to predict docetaxel response in metastatic prostate cancer
Source: Front Oncol. 2023 Jan 16;12:1060864. doi: 10.3389/fonc.2022.1060864 (PMC9885040; doi:10.3389/fonc.2022.1060864)
Supplement: Supplementary file 1 [file DataSheet_1.docx]

**Supplementary Tables**

**Supplementary Table 1. Clinical information of prostate cancer patients.**

| **Sample ID** | **Date of collection** | **mHSPC** | **mCRPC** | **Age** | **GS** | **PSA at sample collection (ng/mL)** | **Treatment(s) before blood collection** | **RECIST response** |
| --- | --- | --- | --- | --- | --- | --- | --- | --- |
| GPB-058 | 16/07/2015 |  | Y | 78 | 3+4=7 | 392 | ADT | SD |
| GPB-069 | 03/02/2015 |  | Y | 65 | 3+4=7 | 313 | ADT | PD |
| GPB-080 | 16.08.2018 |  | Y | 70 | 4+4=8 | 57.23 | Leuproelin until 2018 Bicalutamide added | SD |
| GPB-108 | 18/06/2015 |  | Y | 73 | 4+4=8 | 60 | ADT | SD |
| GPB-119 | 20/08/2015 |  | Y | 67 | 4+5=9 | 23 | ADT | SD |
| GPB-156 | 21/01/2016 |  | Y | 82 | 4+5=9 | 49 | ADT | PD |
| GPB-170 | 29/03/2016 |  | Y | 75 | 4+5=9 | 189 | ADT | SD |
| PPPP111 | 06.07.2017 | Y |  | 89 | 8 | 172 | Degarelix | PD |
| PPPP114 | 13.07.2017 | Y |  | 76 | 9 | 131 | Bicalutamide and Zoladex same day of blood collection | SD |
| PPPP154 | 02.11.2011 | Y |  | 64 | 4+4=8 | 8.5 | ADT | PR |
| PPPP161 | 04.01.2018 | Y |  | 68 | 4+3=7 | 35.46 | Bicalutamide followed by LHRH | PR |
| PPPP166 | 12.10.2017 | Y |  | 72 | 9 | 5.13 | Bicalutamide followed by Leuprorelin in Oct 2018 | SD |
| PPPP261 | 23.09.2018 | Y |  | 70 |  |  | Prednisolone | PR |
| PPPP268 | 06.12.2019 |  | Y | 64 | 5+4=9 | 11.06 | Bicalutamide in 2018 followed by Prostap | PD |
| PTP-005 | 03.11.2016 |  | Y | 60 | 4+4=8 | 7.56 | ADT | PR |
| PTP-007 | 06.01.2017 | Y |  | 65 | 4+3=7 | 2.69 | ADT | PR |
| PTP-009 | 24.01.2017 | Y |  | 76 |  | 42 | ADT | PR |
| PTP-011 | 08.02.2018 |  | Y |  |  | 2031 | Zoladex since 2017 | PD |
| PTP-023 | 25.05.2017 |  | Y | 87 |  | 71 | ADT | SD |
| PTP-028 | 13.07.2017 | Y |  | 77 |  | 18 | ADT | PD |
| PTP-032 | 03.08.2017 | Y |  | 61 | 5+4=9 | 57.39 | Bicalutamide and Prostap | PR |
| PTP-033 | 10.08.2017 | Y |  | 57 | 5+4=9 | 24.52 | Bicalutamide and Prostap | PR |
| PTP-034 | 07.09.2017 |  | Y | 73 |  | 60.65 | ADT | PR |
| PTP-038 | 19.10.2017 | Y |  |  |  | 90.61 | Prostap | PR |
| PTP-040 | 26.10.2017 | Y |  | 65 | 4+4=8 | 31.4 | LHRH agonist (prostap, 26.10.2017 and bicalutamide (casodex) 17.10.17 | PR |
| PTP-041 | 28.11.2017 | Y |  |  | 4+5=8 | 0.74 | Bicalutamide and LHRH injection (prostap) Aug 2017 | PR |
| PTP-042 | 14.11.2017 | Y |  | 69 | 3+4=7 | 10.46 | Leuprorelin Oct 2017 | PD |
| PTP-048 | 07.12.2017 |  | Y | 73 | 4+3=7 | 11.96 | Prednisolone | SD |
| PTP-052 | 11.01.2018 | Y |  | 63 | 4+4=8 | 5.7 | Bicalutamide and Prostap (LHRH) Nov 2017 | PR |
| PTP-053 | 11.01.2018 | Y |  | 71 | N/A | 3.4 | Prostap Oct 2017 | SD |
| PTP-054 | 11.01.2018 | Y |  | 67 | 4+4=8 | 2.79 | Bicalutamide Nov 2017 | SD |
| PTP-056 | 16.01.2018 | Y |  | 68 | 4+5=9 | 0.59 | Degarelix Oct 2017 | PR |
| PTP-057 | 06.03.2018 |  | Y | 60 | 12 | 0.96 | Radical prostatectomy and radiotherapy | PR |
| PTP-063 | 25.01.2018 | Y |  | 73 | 4+5=9 | 1.1 | Bicalutamide Jan 2018 | SD |
| PTP-069 | 08.02.2018 | Y |  | 55 | 4+3=7 |  | LHRH in Nov 2017 | PR |
| PTP-080 | 15.03.2018 | Y |  | 77 | N/A | 6.76 | Prostap Dec 17 | PR |
| PTP-081 | 15.03.2018 |  | Y | 72 | 5+4=9 | 38.14 | ADT | SD |
| PTP-087 | 22.03.2018 | Y |  | 57 | 4+9=13 | 94 | Bicalutamide and Leuprorelin March 18 | SD |
| PTP-093 | 29.03.2018 | Y |  | 53 | 4+5=9 | 1,359 | Degarelix Nov 2017 | PD |
| PTP-109 | 09.05.2018 |  | Y |  |  | 86.1 | LHRH | SD |
| PTP-111 | 10.05.2018 | Y |  | 67 | 4+4=8 | 2.31 | Bicalutamide in April 18 and followed by prostap injection at blood collection May 2018 | PR |
| PTP-120 | 20.06.2018 |  | Y | 69 | 8 | 30.5 | LHRH | PR |
| PTP-124 | 21.06.2018 | Y |  | 79 | 3+4=7 | 56.53 | Bicalutamide followed by Prostap in April 2018 | SD |
| PTP135 | 30.08.2018 | Y |  | 56 | 4+4=8 | 3.73 | Bicalutamide followed by Zoladex in July 2018 | PR |
| PTP138 | 09.08.2018 | Y |  | 48 | 4+5=9 | 0.85 | Bicalutamide followed by LHRH in May 2018 | SD |
| PTP139 | 23.08.2018 | Y |  | 69 | 4+5=9 |  | Started bicalutamide followed by Prostap in Aug 2018 | PD |
| PTP140 | 17.08.2018 | Y |  | 58 | 4+4=8 | 1.83 | Degarelix May 2018 | PD |
| PTP145 | 06.09.2018 | Y |  | 73 | 4+5=9 | 47.98 | Degarelix July 2018 | SD |
| PTP153 | 18.10.2018 | Y |  | 84 |  | 70.78 | Degarelix in July 2018, switched to Prostap and Bicalutamide in Oct 2018 | PR |
| PTP154 | 25.10.2018 |  | Y | 74 | 7 | 13.27 | Gonadorelin and Bicalutamide | PD |
| PTP155 | 01.11.2018 | Y |  | 67 | 4+5=9 | 5.04 | Degarelix Sep 2018 | PR |
| PTP159 | 14.03.2019 | Y |  | 76 | 4+5=9 | 25 | Degarelix Jan 2019 | PD |
| PTP160 | 21.03.2019 | Y |  | 73 |  | 19.64 | Bicalutamide March 2019 | PD |
| PTP161 | 28.03.2019 | Y |  | 68 | 4+5=9 | 34.27 | Bicalutamide followed by prostap March 2019 | SD |
| PTP163 | 02.05.2019 | Y |  | 49 | 3+7=10 | 23 | Bicalutamide | PR |
| PTP164 | 04.07.2019 | Y |  | 71 |  | 11.49 | Degarelix in Sep2018 stopped in Feb2019 as part of intermittent therapy; 2019 started Prostap in view of docetaxel | SD |
| PTP165 | 12.09.2019 | Y |  | 67 | 4+4=8 | 13.12 | Bicalutamide Aug 2019 | PR |
| PTP166 | 24.10.2019 | Y |  | 72 | 4+5=9 | 1.83 | ADT | PR |
| PTP169 | 21.11.2019 | Y |  |  | 4+3=7 | 12.06 | ADT | PR |
| PTP170 | 09.01.2020 | Y |  | 71 | 5+5=10 | 141 | ADT | PR |
| PTP171 | 16.01.2020 | Y |  | 67 | 4+5=9 | 63.53 | ADT | PR |

**Supplementary Table 2. List of TaqMan assays**

| **Gene** | **Assay ID** |
| --- | --- |
| ACKR3 | Hs00664172_s1 |
| ADAMTS1 | Hs00199608_m1 |
| AR | Hs00171172_m1 |
| AXL | Hs01064444_m1 |
| COL5A1 | Hs00609133_m1 |
| FBLN1 | Hs00972609_m1 |
| FOLH1 | Hs00379515_m1 |
| FSTL1 | Hs00907496_m1 |
| GAPDH | Hs02786624_g1 |
| HSPB8 | Hs00205056_m1 |
| IGFBP3 | Hs00181211_m1 |
| KLK2 | Hs00428384_g1 |
| KLK3 | Hs02576345_m1 |
| KLK4 | Hs01549606_g1 |
| LTBP1 | Hs01558763_m1 |
| MCAM | Hs00174838_m1 |
| MRFAP1 | Hs00738144_g1 |
| NANOG | Hs02387400_g1 |
| PLSCR4 | Hs00924823_m1 |
| PODXL | Hs01574644_m1 |
| POU5F1 | Hs04260367_gH |
| PRKD1 | Hs00177037_m1 |
| PROM1 | Hs01009259_m1 |
| PTPRC(CD45) | Hs04189704_m1 |
| SDC2 | Hs00299807_m1 |
| SERPINE2 | Hs00299953_m1 |
| SNAI1 | [Hs00195591_m1](https://www.thermofisher.com/taqman-gene-expression/product/Hs00195591_m1?CID=&ICID=&subtype=) |
| SOX2 | Hs01053049_s1 |
| SPRY4 | Hs00540086_m1 |
| SYDE1 | Hs00973080_m1 |
| TWIST2 | Hs02379973_s1 |
| ZEB1 | Hs01566408_m1 |

**Supplementary Table 3. Prostate cancer specific or docetaxel resistance candidate gene panel with their expression levels in prostate and blood samples and the sources supporting the selection**

| **Gene Name** | **GTEx V7 (TPM)** | | **References** |
| --- | --- | --- | --- |
|  | **Prostate** | **Whole Blood** |  |
| **Genes from literature** | | |  |
| **Prostate specific genes** | | |  |
| **AR** | 22.505 | 0.03073 | [1] |
| ARv7 | #NV | 0.03073 | [2-4] |
| **KLK2** | 2134 | 0.03153 | [5] |
| **KLK3** | 4670.5 | 0.01521 |  |
| **KLK4** | 408.65 | 0 |  |
| **FOLH1** | 50.66 | 0 | [6-8] |
| **Inflammation related genes** | | |  |
| **ACKR3** | 25.14 | 0.9162 | [9, 10] |
| NFKB1 | 24.895 | 20.98 | [11, 12] |
| IL6 | 1.1355 | 0.1398 | [13-16] |
| IL4 | 0.28465 | 0.05439 |  |
| IL8 | 3.7355 | 19.16 |  |
| CCL2 | 79.25 | 0.4507 | [1, 12, 17] |
| C18orf8/MIC1 | 25.325 | 26.15 | [11-13] |
| **(Cancer) signaling pathways** | | |  |
| IGF1R | 23.46 | 8.641 | [18, 19] |
| STAT3 | 99.495 | 105.3 | [12, 20] |
| STAT1 | 39.17 | 35.77 | [21] |
| AKT1 | 72.33 | 28.62 | [1, 22] |
| MAP3K20/ZAK | #NV | #NV | [19] |
| PTCH1 | 6.892 | 0.2611 | [23-25] |
| WNT3 | 2.235 | 0.04663 | [24, 26] |
| **Molecular chaperones** | | |  |
| HSPB1 | 895.1 | 80.93 | [12, 27-30] |
| HSP90AA1 | 354.9 | 130.4 |  |
| HSPA8 | 274.75 | 191 |  |
| HSPA4 | 34.805 | 14.7 |  |
| HSPA5 | 173.35 | 137.7 |  |
| **HSPB8** | 130.2 | 0.3799 |  |
| CLU | 267 | 50.49 | [11, 12] |
| **Cell cycle related genes** | | |  |
| CCNE2 | 1.421 | 0.4788 | [19, 31] |
| DBF4 | 5.597 | 1.825 | [19, 32] |
| KIF11 | 0.8384 | 0.9496 | [33] |
| **Tubulin genes** | | |  |
| TUBB3 | 1.2955 | 0.1293 | [12, 34, 35] |
| TUBB2A | 11.22 | 5.546 |  |
| TUBB6 | 62.22 | 3.424 |  |
| **EMT related genes** | | |  |
| TWIST1 | 4.4635 | 0.04663 | [36-41] |
| **TWIST2** | 18.085 | 0.1756 |  |
| **SNAI1** | 6.5805 | 2.188 |  |
| FOXC2 | 0.727 | 0 |  |
| **ZEB1** | 19.645 | 2.576 |  |
| TGFB1 | 61.995 | 309 | [12, 42] |
| **Stemness related genes** | | |  |
| CD44 | 60.665 | 126 | [1, 43-47] |
| CD24 | #NV | #NV |  |
| **PROM1** | 1.21 | 0.03681 |  |
| **POU5F1** | 5.6895 | 0.4193 |  |
| **SOX2** | 4.6375 | 0.02897 |  |
| ALDH1A1 | 71.915 | 5.128 |  |
| **NANOG** | 0.04525 | 0 |  |
| **Drug efflux pumps** | | |  |
| ABCB1 | 6.395 | 1.145 | [12, 48-50] |
| ABCC2 | 0.82175 | 1.283 |  |
| ABCG2 | 7.642 | 0.2743 |  |
| **Apoptosis related genes** | | |  |
| NGFR | 28.42 | 0.478 | [11, 29, 41, 51, 52] |
| TRAF2 | 15.005 | 5.592 |  |
| TRAF1 | 21.595 | 12.46 |  |
| BIRC2 | 28.69 | 11.33 |  |
| BIRC3 | 7.7375 | 5.338 |  |
| BIRC5 | 0.42 | 0.77 |  |
| BIRC7 | 0.18535 | 0.04038 |  |
| MCL1 | 152.5 | 425.9 |  |
| XIAP | 12.05 | 4.328 |  |
| BCL2 | 11.49 | 2.929 |  |
| BAX | 49 | 70.5 |  |
| BCL2L1 | 76.56 | 147.8 |  |
| BOK | 65.45 | 1.303 |  |
| FAS | 18.58 | 14.96 |  |
| FASLG | 0.26105 | 1.861 |  |
| **Dormancy related genes** | | |  |
| BHLHE41 | 16.23 | 0.3281 | [53, 54] |
| BMP7 | 7.801 | 0.02439 |  |
| DAND5 | 0.1111 | 0.02293 |  |
| NR2F1 | 68.265 | 0.07155 |  |
| **Tumour supressor genes** | | |  |
| BRCA2 | 0.18325 | 0.1414 | [55, 56] |
| **Misc. resistance related genes** | | |  |
| PIM1 | 30.89 | 141.8 | [57, 58] |
| RPN2 | 152.9 | 57.7 | [59, 60] |
| **AXL** | 35.615 | 0.253 | [61, 62] |
| TP53 | 27.195 | 11.51 | [12, 63] |
| SERPINE1 | 19.605 | 2.145 | [24, 64] |
| **IGFBP3** | 88.15 | 0.8901 | [65-67] |
|  |  |  |  |
| **Genes from microarrays** | | | [68, 69] |
| **ACKR3** | 25.525 | 1.064 |  |
| **ADAMTS1** | 97.505 | 0.7032 |  |
| ASS1 | 45.72 | 0.566 |  |
| **COL5A1** | 63.455 | 0.1318 |  |
| **FBLN1** | 292.15 | 0.4643 |  |
| FERMT2 | 29.17 | 0.1688 |  |
| **FSTL1** | 174.9 | 0.9776 |  |
| GEM | 39.32 | 0.1955 |  |
| HEG1 | 15.665 | 1.058 |  |
| **HSPB8** | 137.45 | 0.488 |  |
| **IGFBP3** | 88.15 | 0.8901 |  |
| LAMB1 | 35.52 | 0.373 |  |
| **LTBP1** | 35.3 | 0.672 |  |
| **MCAM** | 81.08 | 0.8092 |  |
| MIR6756 | #NV | #NV |  |
| **PLSCR4** | 18.9 | 0.1942 |  |
| **PODXL** | 29.655 | 0.3615 |  |
| **PRKD1** | 21.225 | 0.0435 |  |
| PROS1 | 13.445 | 0.8088 |  |
| PTPRM | 24.61 | 0.3786 |  |
| RHOBTB3 | 27.41 | 0.48 |  |
| SCARA3 | 40.75 | 0.1224 |  |
| SMAD9 | 18.32 | 0.2314 |  |
| **SYDE1** | 29.435 | 0.09479 |  |
| WDR19 | 18.4 | 1.027 |  |
| WWTR1 | 36.42 | 0.1477 |  |
| ABAT | 12.995 | 3.658 |  |
| ABCB1 | 6.395 | 1.145 |  |
| ABHD3 | 13.58 | 13.77 |  |
| ACOT9 | 19.96 | 13.94 |  |
| ANKRD13C | 12.66 | 1.652 |  |
| ASPH | 18.48 | 7.477 |  |
| ATF3 | 31.75 | 3.128 |  |
| ATG9A | 47.49 | 43.89 |  |
| BNIP3L | 53.955 | 68.06 |  |
| C15orf65 | 4.932 | 0.322 |  |
| C4orf3 | 134.15 | 93.77 |  |
| C5orf42 | 7.2115 | 0.3084 |  |
| CBLB | 10.305 | 3.993 |  |
| CDH12 | 0.022535 | 0 |  |
| CDK19 | 14.92 | 4.686 |  |
| CEMIP | #NV | #NV |  |
| CERK | 23.205 | 32.26 |  |
| CHN1 | 9.93 | 0.3697 |  |
| CITED2 | 50.345 | 32.74 |  |
| CLU | 267 | 50.49 |  |
| COL6A1 | 648.65 | 1.84 |  |
| CREB3L2 | 27.805 | 3.124 |  |
| CRIPT | 14.41 | 6.155 |  |
| CSPP1 | 5.508 | 0.5659 |  |
| CTNND2 | 1.367 | 0.03091 |  |
| CXCR4 | 29.575 | 446.6 |  |
| CYBRD1 | 87.625 | 7.474 |  |
| DPP4 | 28.065 | 2.501 |  |
| DYRK2 | 7.0135 | 2.631 |  |
| CCPG1 | 20.06 | 27.55 |  |
| EML1 | 7.423 | 0.03754 |  |
| EPAS1 | 145.75 | 2.266 |  |
| FAT4 | 3.533 | 0.02785 |  |
| FBN1 | 13.425 | 0.2231 |  |
| FRMD3 | 4.285 | 0.7103 |  |
| FXR1 | 21.305 | 5.569 |  |
| GAB1 | 7.4045 | 1.503 |  |
| GALNS | 17.195 | 14.2 |  |
| GALNT10 | 10.905 | 9.561 |  |
| GHR | 8.4615 | 0.04207 |  |
| GOSR2 | 17.02 | 4.644 |  |
| GRK5 | 23.885 | 9.924 |  |
| GSPT2 | 7.15 | 1.056 |  |
| HDLBP | 157.55 | 32.89 |  |
| HIST1H2BC | 9.3045 | 31.73 |  |
| HIST2H2AA4 | 0.6633 | 1.572 |  |
| HIST2H2BE | 145.9 | 78.64 |  |
| HTRA1 | 90.33 | 2.467 |  |
| ID2 | 89.15 | 41.2 |  |
| IL6R | 12.145 | 59.21 |  |
| ITM2C | 147.3 | 13.38 |  |
| JMJD1C-AS1 | 1.442 | 0.03695 |  |
| KIAA1211 | 0.9535 | 0.05263 |  |
| KLF9 | 73.42 | 6.774 |  |
| KLHL24 | 16.835 | 7.386 |  |
| LONP2 | 31.97 | 7.47 |  |
| SIAH1 | 7.2945 | 1.205 |  |
| LOC153682 | #NV | #NV |  |
| LOX | 6.3005 | 0.08932 |  |
| LPAR3 | 13.55 | 0.05086 |  |
| LPCAT1 | 23.325 | 55.81 |  |
| LRP12 | 4.675 | 0.1948 |  |
| MALAT1 | 462.65 | 117.9 |  |
| MAN1A1 | 20.375 | 16.81 |  |
| MBNL1-AS1 | 4.979 | 0.3501 |  |
| MIB1 | 14.275 | 1.821 |  |
| MIB2 | 55.14 | 13.6 |  |
| MIR612 | #NV | #NV |  |
| NEAT1 | 442.4 | 54.32 |  |
| MLLT11 | 9.2165 | 7.152 |  |
| MUC1 | 41.325 | 1.392 |  |
| MXRA7 | 47.985 | 3.135 |  |
| NAP1L3 | 3.8435 | 0.2546 |  |
| NARF | 25.815 | 55.04 |  |
| NDRG1 | 277.05 | 60.8 |  |
| NEAT1 | 442.4 | 54.32 |  |
| NEFL | 0.28825 | 0.2704 |  |
| NIN | 8.589 | 20.06 |  |
| NOV | 11.135 | 1.023 |  |
| NPR3 | 1.649 | 0.03618 |  |
| NRCAM | 1.152 | 0.06179 |  |
| OSER1-AS1 | 12.37 | 5.506 |  |
| PC | 14.205 | 1.194 |  |
| PCAT6 | 7.456 | 0.3261 |  |
| PDLIM5 | 43.73 | 3.393 |  |
| PDZD8 | 9.5035 | 8.138 |  |
| PGAP1 | 5.468 | 0.1678 |  |
| PGK1 | 78.34 | 234.2 |  |
| PIAS2 | 10.305 | 1.824 |  |
| PLOD1 | 59.12 | 42.31 |  |
| PMP22 | 111.4 | 1.327 |  |
| PPARA | 7.5905 | 0.7702 |  |
| PPP2R2C | 6.318 | 0.0491 |  |
| PSAP | 725.75 | 1430 |  |
| PTPRH | 0.21415 | 0.06325 |  |
| PYGL | 21.35 | 329.8 |  |
| QKI | 11.97 | 8.119 |  |
| QSOX1 | 41.68 | 38.44 |  |
| RAB40B | 13.95 | 0.2817 |  |
| RBM24 | 7.4345 | 0.07231 |  |
| RFX5 | 24.38 | 8.976 |  |
| RGCC | 90.22 | 21.32 |  |
| ROBO1 | 12.53 | 0.04333 |  |
| S100A4 | 148.05 | 991 |  |
| SCD5 | 11.405 | 0.4036 |  |
| **SDC2** | 45.955 | 0.8988 |  |
| SEC14L1 | 19.295 | 71.23 |  |
| SEC24D | 17.44 | 7.692 |  |
| SELM | 262 | 4.575 |  |
| **SERPINE2** | 14.385 | 0.6036 |  |
| SIDT2 | 66.885 | 22.16 |  |
| SLC17A5 | 14.26 | 5.082 |  |
| SLC30A1 | 9.475 | 3.844 |  |
| SLC46A3 | 9.0645 | 9.07 |  |
| SLCO4A1 | 8.507 | 3.115 |  |
| SMAD4 | 27.125 | 5.668 |  |
| SMAD7 | 27.285 | 2.98 |  |
| SMYD2 | 13.08 | 3.232 |  |
| SNAP25 | 8.9605 | 0.2826 |  |
| SPINK1 | 1.258 | 0.2209 |  |
| STC1 | 7.6915 | 0.0657 |  |
| SYNE1 | 11.85 | 4.757 |  |
| TFDP2 | 8.8585 | 2.439 |  |
| TGFBR3 | 21.575 | 1.259 |  |
| THBS1 | 75.11 | 22.25 |  |
| TIMP2 | 221.7 | 56.38 |  |
| TMCO3 | 30.145 | 17.94 |  |
| TMEM165 | 34.78 | 6.363 |  |
| TMEM45A | 9.6195 | 0.6323 |  |
| TMSB15B | 1.493 | 0.08214 |  |
| TNS3 | 17.45 | 1.621 |  |
| TP53INP1 | 34.58 | 19.9 |  |
| TRAF5 | 16.145 | 2.797 |  |
| TRIM9 | 0.78345 | 0.2572 |  |
| TTC14 | 35.545 | 4.066 |  |
| YPEL2 | 13.78 | 6.14 |  |
| ZNF500 | 11.465 | 2.155 |  |

Genes included in final gene panel are shown in **bold**. Abbreviations: GETx, Genotype-Tissue Expression; TPM, transcripts per million.

References

1. Seruga, B., A. Ocana, and I.F. Tannock, *Drug resistance in metastatic castration-resistant prostate cancer.* Nat Rev Clin Oncol, 2011. **8**(1): p. 12-23.

2. Liu, L., et al., *Calpain and AR-V7: Two potential therapeutic targets to overcome acquired docetaxel resistance in castration-resistant prostate cancer cells.* Oncol Rep, 2017. **37**(6): p. 3651-3659.

3. Marin, M., et al., *ARV7 and ARFL mRNA in blood to predict androgen receptor inhibitors and docetaxel response in castration-resistant prostate cancer patients.* Journal of Clinical Oncology, 2019. **37**(7_suppl): p. 207-207.

4. Okegawa, T., et al., *AR-V7 in circulating tumor cells cluster as a predictive biomarker of abiraterone acetate and enzalutamide treatment in castration-resistant prostate cancer patients.* Prostate, 2018. **78**(8): p. 576-582.

5. Emami, N. and E.P. Diamandis, *Utility of kallikrein-related peptidases (KLKs) as cancer biomarkers.* Clin Chem, 2008. **54**(10): p. 1600-7.

6. Chang, S.S., *Overview of prostate-specific membrane antigen.* Reviews in urology, 2004. **6 Suppl 10**(Suppl 10): p. S13-S18.

7. Nagaya, N., et al., *Prostate-specific membrane antigen in circulating tumor cells is a new poor prognostic marker for castration-resistant prostate cancer.* PLOS ONE, 2020. **15**(1): p. e0226219.

8. Bravaccini, S., et al., *PSMA expression: a potential ally for the pathologist in prostate cancer diagnosis.* Sci Rep, 2018. **8**(1): p. 4254.

9. Zhu, S., et al., *Expression profile-based screening for critical genes reveals S100A4, ACKR3 and CDH1 in docetaxel-resistant prostate cancer cells.* Aging (Albany NY), 2019. **11**(24): p. 12754-12772.

10. Rani, A., P. Dasgupta, and J.J. Murphy, *Prostate Cancer: The Role of Inflammation and Chemokines.* The American Journal of Pathology, 2019. **189**(11): p. 2119-2137.

11. O'Neill, A.J., et al., *Characterisation and manipulation of docetaxel resistant prostate cancer cell lines.* Mol Cancer, 2011. **10**: p. 126.

12. Magadoux, L., et al., *Emerging targets to monitor and overcome docetaxel resistance in castration resistant prostate cancer (review).* Int J Oncol, 2014. **45**(3): p. 919-28.

13. Mahon, K.L., et al., *Cytokine profiling of docetaxel-resistant castration-resistant prostate cancer.* Br J Cancer, 2015. **112**(8): p. 1340-8.

14. Domingo-Domenech, J., et al., *Interleukin 6, a Nuclear Factor-κB Target, Predicts Resistance to Docetaxel in Hormone-Independent Prostate Cancer and Nuclear Factor-κB Inhibition by PS-1145 Enhances Docetaxel Antitumor Activity.* Clinical Cancer Research, 2006. **12**(18): p. 5578-5586.

15. Harshman, L.C., et al., *Impact of baseline serum IL-8 on metastatic hormone-sensitive prostate cancer outcomes in the Phase 3 CHAARTED trial (E3805).* Prostate, 2020. **80**(16): p. 1429-1437.

16. Horvath, L., et al., *The role of macrophages in docetaxel (DTX) resistance in castrate-resistant prostate cancer (CRPC).* Journal of Clinical Oncology, 2013. **31**(15_suppl): p. e22175-e22175.

17. Qian, D.Z., et al., *CCL2 is induced by chemotherapy and protects prostate cancer cells from docetaxel-induced cytotoxicity.* The Prostate, 2010. **70**(4): p. 433-442.

18. Zhang, D., et al., *Regulation of SOD2 and β-arrestin1 by interleukin-6 contributes to the increase of IGF-1R expression in docetaxel resistant prostate cancer cells.* European Journal of Cell Biology, 2014. **93**(7): p. 289-298.

19. Lee, S., et al., *Analysis of resistance-associated gene expression in docetaxel-resistant prostate cancer cells.* Oncology letters, 2017. **14**(3): p. 3011-3018.

20. Hu, F., et al., *Docetaxel-mediated autophagy promotes chemoresistance in castration-resistant prostate cancer cells by inhibiting STAT3.* Cancer Lett, 2018. **416**: p. 24-30.

21. Patterson, S.G., et al., *Novel role of Stat1 in the development of docetaxel resistance in prostate tumor cells.* Oncogene, 2006. **25**(45): p. 6113-6122.

22. Dey, G., et al., *Resensitization of Akt Induced Docetaxel Resistance in Breast Cancer by ‘Iturin A’ a Lipopeptide Molecule from Marine Bacteria Bacillus megaterium.* Scientific Reports, 2017. **7**(1): p. 17324.

23. Mimeault, M., et al., *Inhibition of hedgehog signaling improves the anti-carcinogenic effects of docetaxel in prostate cancer.* Oncotarget, 2015. **6**(6): p. 3887-3903.

24. Lee, S., et al., *Analysis of resistance‑associated gene expression in docetaxel‑resistant prostate cancer cells.* Oncol Lett, 2017. **14**(3): p. 3011-3018.

25. Hasanovic, A. and I. Mus-Veteau, *Targeting the Multidrug Transporter Ptch1 Potentiates Chemotherapy Efficiency.* Cells, 2018. **7**(8): p. 107.

26. Kumar, V., et al., *The Role of Notch, Hedgehog, and Wnt Signaling Pathways in the Resistance of Tumors to Anticancer Therapies.* Frontiers in Cell and Developmental Biology, 2021. **9**(857).

27. O'Connell, K., et al., *The use of LC-MS to identify differentially expressed proteins in docetaxel-resistant prostate cancer cell lines.* Proteomics, 2012. **12**(13): p. 2115-26.

28. Ku, S., et al., *Inhibition of Hsp90 augments docetaxel therapy in castrate resistant prostate cancer.* PLoS One, 2014. **9**(7): p. e103680.

29. Desarnaud, F., et al., *Gene expression profiling of the androgen independent prostate cancer cells demonstrates complex mechanisms mediating resistance to docetaxel.* Cancer biology & therapy, 2011. **11**(2): p. 204-212.

30. Cristofani, R., et al., *Dual role of autophagy on docetaxel-sensitivity in prostate cancer cells.* Cell death & disease, 2018. **9**(9): p. 889-889.

31. Handle, F., et al., *Drivers of AR indifferent anti-androgen resistance in prostate cancer cells.* Scientific Reports, 2019. **9**(1): p. 13786.

32. Lombard, A.P., et al., *Activation of the <em>ABCB1</em> Amplicon in Docetaxel- and Cabazitaxel-Resistant Prostate Cancer Cells.* Molecular Cancer Therapeutics, 2021. **20**(10): p. 2061-2070.

33. Jiang, M., et al., *KIF11 is required for proliferation and self-renewal of docetaxel resistant triple negative breast cancer cells.* Oncotarget, 2017. **8**(54): p. 92106-92118.

34. Shalli, K., et al., *Alterations of beta-tubulin isotypes in breast cancer cells resistant to docetaxel.* Faseb j, 2005. **19**(10): p. 1299-301.

35. Maahs, L., et al., *Class III β-tubulin expression as a predictor of docetaxel-resistance in metastatic castration-resistant prostate cancer.* PloS one, 2019. **14**(10): p. e0222510-e0222510.

36. Hanrahan, K., et al., *The role of epithelial–mesenchymal transition drivers ZEB1 and ZEB2 in mediating docetaxel-resistant prostate cancer.* Molecular Oncology, 2017. **11**(3): p. 251-265.

37. Marín-Aguilera, M., et al., *Epithelial-to-mesenchymal transition mediates docetaxel resistance and high risk of relapse in prostate cancer.* Mol Cancer Ther, 2014. **13**(5): p. 1270-84.

38. Zhang, G., et al., *miR-27b and miR-34a enhance docetaxel sensitivity of prostate cancer cells through inhibiting epithelial-to-mesenchymal transition by targeting ZEB1.* Biomedicine & Pharmacotherapy, 2018. **97**: p. 736-744.

39. Ashrafizadeh, M., et al., *New insight towards development of paclitaxel and docetaxel resistance in cancer cells: EMT as a novel molecular mechanism and therapeutic possibilities.* Biomedicine & Pharmacotherapy, 2021. **141**: p. 111824.

40. Ren, J., et al., *Inhibition of ZEB1 reverses EMT and chemoresistance in docetaxel-resistant human lung adenocarcinoma cell line.* Journal of Cellular Biochemistry, 2013. **114**(6): p. 1395-1403.

41. Ippolito, L., et al., *Metabolic shift toward oxidative phosphorylation in docetaxel resistant prostate cancer cells.* Oncotarget, 2016. **7**(38): p. 61890-61904.

42. Li, Y., et al., *TGF-β causes Docetaxel resistance in Prostate Cancer via the induction of Bcl-2 by acetylated KLF5 and Protein Stabilization.* Theranostics, 2020. **10**(17): p. 7656-7670.

43. Wróbel, T., et al., *CD44+ cells determine fenofibrate-induced microevolution of drug-resistance in prostate cancer cell populations.* STEM CELLS, 2020. **38**(12): p. 1544-1556.

44. Canesin, G., et al., *STAT3 inhibition with galiellalactone effectively targets the prostate cancer stem-like cell population.* Scientific Reports, 2020. **10**(1): p. 13958.

45. Landen, C.N., Jr., et al., *Targeting aldehyde dehydrogenase cancer stem cells in ovarian cancer.* Molecular cancer therapeutics, 2010. **9**(12): p. 3186-3199.

46. Zhang, X., et al., *Docetaxel promotes cell apoptosis and decreases SOX2 expression in CD133‑expressing hepatocellular carcinoma stem cells by suppressing the PI3K/AKT signaling pathway.* Oncol Rep, 2019. **41**(2): p. 1067-1074.

47. Qiu, X., et al., *Targeting Ezh2 could overcome docetaxel resistance in prostate cancer cells.* BMC cancer, 2019. **19**(1): p. 27-27.

48. Zhu, Y., et al., *Inhibition of ABCB1 expression overcomes acquired docetaxel resistance in prostate cancer.* Mol Cancer Ther, 2013. **12**(9): p. 1829-36.

49. Lombard, A.P., et al., *ABCB1 Mediates Cabazitaxel-Docetaxel Cross-Resistance in Advanced Prostate Cancer.* Mol Cancer Ther, 2017. **16**(10): p. 2257-2266.

50. Nakanishi, T. and D.D. Ross, *Breast cancer resistance protein (BCRP/ABCG2): its role in multidrug resistance and regulation of its gene expression.* Chinese journal of cancer, 2012. **31**(2): p. 73-99.

51. De Angelis, M.L., et al., *Stem Cell Plasticity and Dormancy in the Development of Cancer Therapy Resistance.* Frontiers in Oncology, 2019. **9**(626).

52. Campbell, K.J., et al., *MCL-1 is a prognostic indicator and drug target in breast cancer.* Cell Death & Disease, 2018. **9**(2): p. 19.

53. Mitra, A., L. Mishra, and S. Li, *EMT, CTCs and CSCs in tumor relapse and drug-resistance.* Oncotarget, 2015. **6**(13): p. 10697-711.

54. Chung, J.-S., et al., *PD71-07 GAS6, KLK2, AND BMP7 DETECTED IN CIRCULATING TUMOR CELLS PREDICT RESISTANCE TO CHEMOTHERAPY IN MCRPC.* Journal of Urology, 2017. **197**(4S): p. e1358-e1358.

55. Nientiedt, C., et al., *Mutations in BRCA2 and taxane resistance in prostate cancer.* Sci Rep, 2017. **7**(1): p. 4574.

56. Egawa, C., et al., *Decreased expression of BRCA2 mRNA predicts favorable response to docetaxel in breast cancer.* International Journal of Cancer, 2001. **95**(4): p. 255-259.

57. Holder, S.L. and S.A. Abdulkadir, *PIM1 kinase as a target in prostate cancer: roles in tumorigenesis, castration resistance, and docetaxel resistance.* Curr Cancer Drug Targets, 2014. **14**(2): p. 105-14.

58. Zemskova, M., et al., *The PIM1 kinase is a critical component of a survival pathway activated by docetaxel and promotes survival of docetaxel-treated prostate cancer cells.* The Journal of biological chemistry, 2008. **283**(30): p. 20635-20644.

59. Fujimoto, D., et al., *RPN2 is effective biomarker to predict the outcome of combined chemotherapy docetaxel and cisplatin for advanced gastric cancer.* Oncotarget, 2018. **9**(20): p. 15208-15218.

60. *RPN2 overexpression confers resistance to docetaxel in breast cancer.* Nature Clinical Practice Oncology, 2008. **5**(12): p. 686-686.

61. Wilson, C., et al., *AXL Inhibition Sensitizes Mesenchymal Cancer Cells to Antimitotic Drugs.* Cancer Research, 2014. **74**(20): p. 5878-5890.

62. Lin, J.Z., et al., *Targeting AXL overcomes resistance to docetaxel therapy in advanced prostate cancer.* Oncotarget, 2017. **8**(25): p. 41064-41077.

63. Liu, C., et al., *Functional p53 determines docetaxel sensitivity in prostate cancer cells.* The Prostate, 2013. **73**(4): p. 418-427.

64. Zhang, Q., L. Lei, and D. Jing, *Knockdown of SERPINE1 reverses resistance of triple‑negative breast cancer to paclitaxel via suppression of VEGFA.* Oncol Rep, 2020. **44**(5): p. 1875-1884.

65. Denduluri, S.K., et al., *Insulin-like growth factor (IGF) signaling in tumorigenesis and the development of cancer drug resistance.* Genes & Diseases, 2015. **2**(1): p. 13-25.

66. Liu, L. and X. Li, *A Review of IGF1 Signaling and IGF1-related Long Noncoding RNAs in Chemoresistance of Cancer.* Current Cancer Drug Targets, 2020. **20**(5): p. 325-334.

67. Uzoh, C.C., et al., *Insulin-like growth factor-binding protein-2 promotes prostate cancer cell growth via IGF-dependent or -independent mechanisms and reduces the efficacy of docetaxel.* British Journal of Cancer, 2011. **104**(10): p. 1587-1593.

68. Domingo-Domenech, J., et al., *Suppression of acquired docetaxel resistance in prostate cancer through depletion of notch- and hedgehog-dependent tumor-initiating cells.* Cancer Cell, 2012. **22**(3): p. 373-88.

69. Marín-Aguilera, M., et al., *Identification of docetaxel resistance genes in castration-resistant prostate cancer.* Mol Cancer Ther, 2012. **11**(2): p. 329-39.
